# Use of ferrous iron by metallo-β-lactamases

**DOI:** 10.1016/j.jinorgbio.2016.07.013

**Published:** 2016-10

**Authors:** Samuel T. Cahill, Hanna Tarhonskaya, Anna M. Rydzik, Emily Flashman, Michael A. McDonough, Christopher J. Schofield, Jürgen Brem

**Affiliations:** Chemistry Research Laboratory, Oxford, United Kingdom

**Keywords:** MBL, metallo-β-lactamase, SBL, serine-β-lactamase, BcII, metallo-β-lactamase II from *Bacillus cereus*, VIM-2, Verona integron-encoded metallo-β-lactamase 2, CPSF, cleavage/polyadenylation specificity factor, SNM, sensitivity to nitrogen mustard, ROO, rubredoxin:oxygen reductase, ETHE1, ethylmalonic encephalopathy 1, Pce, phosphorylcholine esterase, NDM-1, New Delhi metallo-β-lactamase 1, IPTG, isopropyl β-D-1-thiogalactopyranoside, PMSF, phenylmethylsulfonyl fluoride, SDS-PAGE, sodium dodecyl sulfate polyacrylamide gel electrophoresis, EDTA, ethylenediaminetetraacetic acid, TCEP·HCl, tris(2-carboxyethyl)phosphine hydrochloride salt, IC_50_, half maximal inhibitory concentration, ESI MS, electrospray ionisation mass spectrometry, Antibiotic resistance, β-Lactam antibiotics, Metallo-β-lactamase, Zinc hydrolase, Metalloenzyme, Carbapenem

## Abstract

Metallo-β-lactamases (MBLs) catalyse the hydrolysis of almost all β-lactam antibacterials including the latest generation carbapenems and are a growing worldwide clinical problem. It is proposed that MBLs employ one or two zinc ion cofactors *in vivo*. Isolated MBLs are reported to use transition metal ions other than zinc, including copper, cadmium and manganese, with iron ions being a notable exception. We report kinetic and biophysical studies with the di-iron(II)-substituted metallo-β-lactamase II from *Bacillus cereus* (di-Fe(II) BcII) and the clinically relevant B1 subclass Verona integron-encoded metallo-β-lactamase 2 (di-Fe(II) VIM-2). The results reveal that MBLs can employ ferrous iron in catalysis, but with altered kinetic and inhibition profiles compared to the zinc enzymes. A crystal structure of di-Fe(II) BcII reveals only small overall changes in the active site compared to the di-Zn(II) enzyme including retention of the di-metal bridging water; however, the positions of the metal ions are altered in the di-Fe(II) compared to the di-Zn(II) structure. Stopped-flow analyses reveal that the mechanism of nitrocefin hydrolysis by both di-Fe(II) BcII and di-Fe(II) VIM-2 is altered compared to the di-Zn(II) enzymes. Notably, given that the MBLs are the subject of current medicinal chemistry efforts, the results raise the possibility the Fe(II)-substituted MBLs may be of clinical relevance under conditions of low zinc availability, and reveal potential variation in inhibitor activity against the differently metallated MBLs.

## Highlights

• Metallo-β-lactamases (MBLs) can employ ferrous iron to hydrolyse β-lactam antibiotics.
• Pre-steady state kinetics reveals altered mechanistic profiles of iron-substituted MBLs.
• Di-Fe(II) MBL from *Bacillus cereus* shows similar active site geometry to di-Zn(II) MBLs.
• Zinc(II)- and iron(II)-bound MBLs can exhibit different susceptibilities to inhibitors.

## Graphical abstract

Metallo-β-lactamases (MBLs) catalyse the hydrolysis of almost all β-lactam antibiotics and are a growing clinical problem. It is proposed that MBLs employ one or two zinc ion cofactors. We report that MBLs can employ ferrous iron, but with altered kinetic and inhibition profiles compared to the zinc enzymes.

**Figure:**
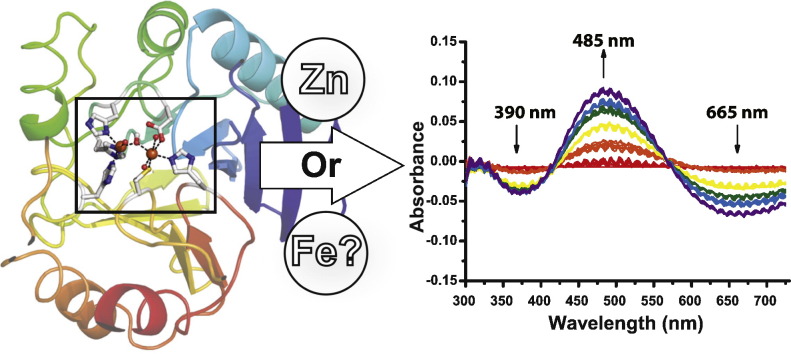

## 1 Introduction

**Fig. 1:**
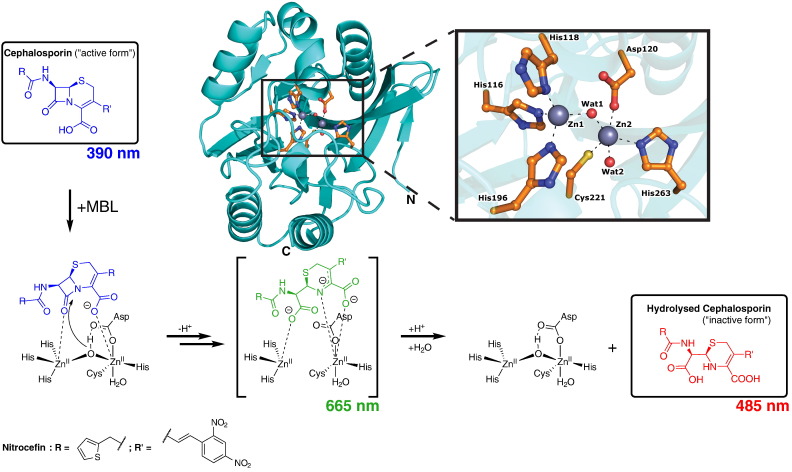
Outline catalytic mechanism for the B1 subclass MBLs illustrated with a cephalosporin substrate. Hydrolysis is proposed to occur via nucleophilic attack of a di-Zn(II)-bridging water/hydroxide onto the β-lactam ring carbonyl and likely proceeds through a tetrahedral intermediate (not shown). Depending on the substrate-enzyme combination, evidence for an anionic intermediate (shown in parentheses) is sometimes observed. Numbers indicate the absorbance wavelength λ_max_ of the corresponding assigned species present during the hydrolysis of nitrocefin. The protein image is a view from a structure of the B1 MBL BcII (from *B. cereus*, PDB accession code: 4C09) [16]. The di-Zn(II) ion-binding enzyme exhibits two metal binding sites: Zn1 is coordinated by His116, His118 and His196 at site 1, while Zn2 is coordinated by Asp120, Cys221 and His263 at site 2. Wat1 bridges the two metal centres while a second apical water, Wat2, coordinates Zn2. The protonation states of the waters are uncertain, though it is proposed that a metal-bound hydroxide acts as the nucelophile.

More than 70 years after their first clinical application, the β-lactams remain the most important antibacterials in use [1]. β-Lactamases constitute an important mode of resistance to β-lactam antibacterials by catalysing hydrolysis of the β-lactam ring to give inactive β-amino acid products [2], [3], [4], [5]. In mechanistic terms, β-lactamases are divided into two classes, i.e. those that employ a nucleophilic serine residue (serine β-lactamases (SBLs), Ambler classes A, C, and D) and those requiring metal ions for hydrolysis (metallo-β-lactamases (MBLs), Ambler class B) [6], [7]. The MBLs pose an important threat to the continued use of β-lactam antibacterials through their ability to hydrolyse almost all known types, with the sole exception of the monobactams [8], [9]. The metallo-β-lactamase II from *Bacillus cereus* (BcII) was the first MBL for which a crystal structure was solved; this structure revealed the MBL fold as a hitherto unrecognised and widely distributed metal-binding enzyme superfamily [10]. The true MBLs, i.e. those catalysing β-lactam hydrolysis, are further divided into three subclasses, B1, B2, and B3. Both B1 and B3 enzymes bind two Zn(II) ions in their native state, with the exception of the B3 enzyme GOB, which can exhibit activity when a single Zn(II) ion is bound [11], whereas B2 enzymes bind one Zn(II) ion and are inhibited through binding of a second ion [12], [13]. The active site of the B1 MBLs is characterised by two zinc coordination sites. One zinc ion, Zn1, is coordinated by three histidine residues, His116, His118 and His196, constituting Site 1; the other, Zn2, is coordinated by an aspartate, cysteine and histidine trio of ligands, Asp120, Cys221 and His263, constituting Site 2 (BBL numbering scheme used throughout [14]). A water molecule, proposed to be a hydroxide ion, Wat1, bridges the two metal centres while an additional ‘terminal’ water molecule, Wat2, is bound to Zn2 (Fig. 1) [15].

The true MBLs exhibit promiscuity in transition metal binding and the alternatively metallated states can retain catalytic activity [17], [18], [19], [20]. The promiscuous metal binding of BcII has been demonstrated through the restoration of catalytic activity to the apo-enzyme by the addition of cadmium(II), cobalt(II) or manganese(II) salts, which manifest varying levels of catalytic efficiency [17]. Co(II)-substituted BcII and Verona integron-encoded metallo-β-lactamase 2 (VIM-2) have been utilised in stopped-flow assays [17], [20], [21] in which anionic intermediates have been characterised [20], [21]. Furthermore a class B3 MBL, L1, is active with a di-Cu(II)-bound centre [18]. A notable exception are iron ions; although the activity of an assigned FeZn L1 enzyme has been demonstrated [22], no or negligible levels of activity have been reported with enzymes binding solely iron as a cofactor [11], [23], [24].

In addition to the true MBLs, MBL fold proteins constitute a superfamily whose members exhibit diversity in both their metal binding preferences and their catalytic functionality [13]. MBL fold proteins include di-zinc MBL fold enzymes acting on small-molecule substrates such as glyoxalase II, methyl parathion hydrolase from *Pseudomonas sp.*, and *N*-acyl homoserine lactone hydrolase from *Bacillus thuringensis* [25], [26], [27]. A further subset of MBL fold enzymes is involved in RNA processing and DNA repair, for example tRNA ribonuclease Z from *E. coli* and the human cleavage/polyadenylation specificity factor, CPSF73, and DNA cross-link repair protein, sensitive to nitrogen mustard, SNM1 [28], [29], [30]. The MBL fold is able to accommodate both mono- and di-ferrous iron binding. Iron-binding MBL fold proteins can exhibit oxidoreductase or hydrolase activities; examples of MBL fold proteins catalysing oxidoreductase reactions include the di-iron rubredoxin:oxygen reductase (ROO) from *Desulfovibrio gigas* and FprA from *Moorella thermoacetica* as well as the mono-iron dioxygenase ethylmalonic encephalopathy 1 (ETHE1) enzyme which is involved in the oxidative degradation of H_2_S [31], [32], [33], [34]. A recent study has also revealed that the phosphorylcholine esterase (Pce) from *Streptococcus pneumoniae* is an interesting example of an MBL-fold hydrolase employing a di-Fe(II)-bound active site [35].

There are reported instances of both true MBLs and MBL fold proteins co-purifying with iron [26], [28], [36]; however, the true MBLs have been reported, by several groups, as being inactive with solely iron ions [11], [24], [37]. These observations are interesting given the role of iron availability in bacterial pathogenicity and that both di- and mono-Fe(II)-bound MBL fold enzymes, and in particular the di-Fe(II) hydrolase Pce, have been characterised as being active [35], [38]. Herein we report that true MBLs reconstituted specifically with Fe(II) show activity against the reporter substrate nitrocefin as well as the clinically used antibiotic meropenem. A crystal structure of di-Fe(II) BcII reveals only small changes in the active site compared to the di-Zn(II) enzyme; amino acid side chains of the di-Fe(II) active site are superimposable on those of a di-Zn(II) structure and the bridging water is retained, however the positions of the Fe(II) ions are altered. Stopped-flow analyses imply that the mechanism of nitrocefin hydrolysis by both di-Fe(II) BcII and di-Fe(II) VIM-2 is altered compared to the di-Zn(II) enzymes. Importantly, given that the MBLs are the subject of current medicinal chemistry efforts, we demonstrate the potential for variation in inhibitor activity against differently metallated species, i.e. Zn(II) and Fe(II). The results have implications for the design of MBL inhibitors and raise questions about the use of specific metal ions by MBL proteins in a cellular context.

## 2 Materials & methods

### 2.1 Enzyme production

Recombinant BcII protein was produced in *E. coli* BL21(DE3)pLysS cells grown at 37 °C using 2TY medium supplemented with 50 μg mL^− 1^ ampicillin and 34 μg mL^− 1^ chloramphenicol, as was previously reported [39]. The cells were grown until an OD_600_ of 0.6–0.7 was reached and induced with 0.5 mM isopropyl β-D-1-thiogalactopyranoside (IPTG). The cells were grown for a further 4 h after induction. Cells were harvested by centrifugation (10 min, 10,000*g*), resuspended in 50 mL Buffer A (20 mM MES pH 6.35) supplemented with 0.2 mM ZnCl_2_, DNAse I and phenylmethylsulfonyl fluoride (PMSF), then lysed by sonication. The lysate was loaded onto an SP Sepharose column (1.5 × 12 cm with a 25 mL bed volume), which had been pre-equilibrated with Buffer A. Bound proteins were eluted with a 0–1 M NaCl gradient in Buffer A. The purity of the fractions was determined using SDS-PAGE analysis and those fractions containing highly purified BcII (> 95% pure by SDS-PAGE) were concentrated by centrifugal ultrafiltration. Concentrated BcII was rediluted into 50 mL Buffer A and purified a second time on the SP Sepharose column using the same protocol. Fractions containing purified BcII were concentrated by centrifugal ultrafiltration and the concentration of enzyme determined using a NanoDrop spectrophotometer (Thermo Scientific, ε = 29,450 M^− 1^ cm^− 1^).

Recombinant VIM-2 with an *N*-terminal His-tag was produced in *E. coli* BL21(DE3)pLysS cells at 37 °C using 2TY medium supplemented with 50 μg mL^− 1^ ampicillin and 34 μg mL^− 1^ chloramphenicol as previously reported [40]. Cells were grown until an OD_600_ of 0.6–0.7 was reached. At this point the temperature was lowered to 30 °C and expression was induced with IPTG (0.5 mM final concentration). The cells were incubated for a further 4 h at this temperature. Cells were harvested by centrifugation (10 min, 10,000*g*), resuspended in 50 mL lysis buffer (50 mM Tris pH 7.5, 500 mM NaCl, 0.2% Tween 20, 5 mM imidazole) supplemented with DNAse I, lysosyme and EDTA-free protease-inhibitor cocktail and lysed by sonication. The supernatant was loaded onto a 5 mL HisTrap HP column followed by extensive washing with 50 mM Tris pH 7.5, 500 mM NaCl, 5 mM imidazole before elution with a 20–500 mM imidazole gradient. Fractions containing the purified VIM-2 were concentrated by centrifugal ultrafiltration. The resultant protein solution was injected onto a Superdex S200 column (300 mL) and eluted with 20 mM Tris pH 7.5, 200 mM NaCl. To produce untagged enzyme, fractions containing pure His-tagged VIM-2 were incubated overnight at 4 °C with His-tagged 3C protease (1:100 w/w) to remove the *N*-terminal His-tag. In order to remove the 3C protease together with any uncleaved protein the digestion mixture was purified using a second HisTrap HP column pre-equilibrated in 50 mM Tris pH 7.5, 500 mM NaCl, 20 mM imidazole. The active and purified enzyme fractions as identified by SDS-PAGE and a nitrocefin-based activity measurement, were pooled and concentrated by centrifugal ultrafiltration. The concentrations of the purified proteins were determined using a NanoDrop spectrophotometer (Thermo Scientific, ε = 31,400 M^− 1^ cm^− 1^).

### 2.2 Generation of apo-enzymes

Apo-BcII and apo-VIM-2 were generated using an adaptation of a previous method [17]. Thus, di-Zn(II) binding enzyme solutions were dialysed against three changes of > 100 volumes of an EDTA-containing solution (50 mM HEPES pH 7.5, 200 mM NaCl, 20 mM EDTA, 2 mM TCEP·HCl) over 24 h. EDTA was removed by a second dialysis of three changes of > 100 volumes of a metal-free solution (50 mM HEPES pH 7.5, 100 mM NaCl, 2 mM TCEP·HCl, Chelex 100) over 24 h. All dialyses were carried out at 4 °C using Slide-A-Lyzer® Dialysis Cassettes (Thermo Scientific). The concentrations of the apo-enzymes were determined using a NanoDrop spectrophotometer measuring absorption at 280 nm (ε = 29,450 M^− 1^ cm^− 1^ and ε = 31,400 M^− 1^ cm^− 1^ for BcII and VIM-2, respectively). All subsequent concentrations used in assays were based on this measurement.

### 2.3 Electrospray ionisation mass spectrometry

The MS data were acquired using a Q-TOF mass spectrometer (Q-TOF micro, Micromass, Altrincham, U.K.) interfaced with a NanoMate (Advion Biosciences, Ithaca, NY) with a chip voltage of 1.7 kV and a delivery pressure 0.5 psi. The sample cone voltage was 50 V with a source temperature of 100 °C. The pressure at the interface between the atmospheric source and the high vacuum region was fixed at 6.30 mbars. Data were processed using MassLynx 4.0 (Waters).

### 2.4 Stopped-flow UV/Vis absorption kinetics

All metal solutions and metal-substituted enzymes for stopped-flow kinetics were prepared at ≤ 40 ppm O_2_ using deoxygenated buffers. Buffers used were: 50 mM HEPES pH 7.5, 200 mM NaCl; 50 mM MES pH 6.5, 200 mM NaCl; 50 mM MES pH 5.5, 200 mM NaCl. All buffers were treated with Chelex 100 to remove residual metal ions. Aliquots of apo-enzyme were diluted to 100 μM by addition of reaction buffer (1 mL final volume). The enzyme was incubated with two equivalents of *tris*(2-carboxyethyl)phosphine hydrochloride salt (TCEP·HCl), by addition of 2 μL of a 100 mM stock, for 5 min. Metal-substituted enzymes were then produced by the addition of 3.5 equivalents of metal salt to the apo-enzyme (3.5 μL of a 100 mM stock in buffer). 100 μM Metal-substituted enzyme was rapidly mixed with 100 μM nitrocefin solution in a 1:1 ratio at 5 °C. Spectra were recorded over 100 s using a photodiode-array detector. Subsequent analysis of kinetic traces was carried out using Origin 8.5 [41]. Curves corresponding to substrate depletion (390 nm) and product accumulation (485 nm) were fitted by a single exponential function, whilst 665 nm features were fitted by a double exponential function.

### 2.5 Steady-state kinetics

Steady-state kinetic studies were carried out using a Pherastar FS microplate reader and UV-Star 96 well clear microplates (Greiner Bio-One). Samples of 1 mM apo-enzyme were pre-incubated with two equivalents of TCEP·HCl for 5 min prior to addition of 3.5 equivalents of the desired metal before dilution to concentrations required for kinetic assays. For Fe(II)-substituted enzymes, plates were prepared at ≤ 2 ppm O_2_ using deoxygenated buffer and sealed prior to absorbance readings. Note, although we can't rule out trace contamination by other metals (including Zn(II)) in the case of Fe(II) MBLs (and vice versa), the different properties of the Fe(II)- and Zn(II)-substituted MBLs imply the assigned metallated states as being predominant. Reaction progress at room temperature was followed by monitoring changes in absorbance at 485 nm and 295 nm, for nitrocefin and meropenem respectively [40], [42]. Enzyme concentrations suitable for kinetic measurements were obtained by monitoring changes in absorbance for a range of enzyme concentrations between 30 nM and 30 pM, these are shown in Table 1 & Table S1. Kinetic constants were determined using the initial rate of hydrolysis (nitrocefin product formation or meropenem substrate depletion) with the reaction initiated through the addition of enzyme to pre-prepared substrate concentrations. Substrate concentrations ranged between 400 μM and 0.4 μM. The Michaelis-Menten equation was fitted to data by non-linear regression using GraphPad Prism software to calculate the Michaelis-Menten constant and the limiting rate [43].

### 2.6 Inhibition assays

Inhibition assays were carried out using nitrocefin as a reporter substrate [40]. Enzyme concentrations were the same as those employed in equilibrium kinetic studies. Nitrocefin was used at a concentration of 10 μM. IC_50_ values were determined by pre-incubating the enzyme with the inhibitor in the assay buffer at 24 °C for 5 min prior to the addition of substrate, as performed in previous studies [40], [42]. Residual enzyme activity was determined for a range of inhibitor concentrations. Non-linear regression fitting of IC_50_ curves was carried out using a three-parameter dose-response curve in GraphPad Prism [43]. Errors are expressed as: $\frac{\sigma \left(\log{\mathrm{IC}}_{50}\right)}{\log{\mathrm{IC}}_{50}}\times {\mathrm{IC}}_{50}$.

### 2.7 Crystallisation, X-ray data collection and processing

All solutions were deoxygenated and preparation of samples and plates was carried out inside a glovebox (Belle Technology) at < 2 ppm oxygen. A 24 mg mL^− 1^ solution of apo-BcII in Chelex 100-treated 50 mM HEPES pH 7.5, 100 mM NaCl was supplemented with 2 mM TCEP·HCl and 16 mM iron(II) ammonium sulfate. Crystallisation was performed at room temperature using sitting drop vapour diffusion methods in Cryschem 24 well plates (Hampton Research) sealed with Crystal Clear sealing tape (Hampton Research). Crystals were obtained in 23% PEG 3350, 0.10 M ammonium sulfate, 0.1 M Bis-Tris pH 5.5 using a 1:1 mixture of protein to well solution. Crystals appeared over 24 h. Crystals were cryo-cooled using 25% glycerol in well solution as a cryo-protectant. Diffraction data were collected at 100 K on beamline I02 of the Diamond Light Source, Didcot. Diffraction data were integrated and scaled using iMosflm and AIMLESS [44], [45] followed by iterative rounds of refinement and model fitting using PHENIX and Coot [46], [47].

## 3 Results

### 3.1 Steady-state kinetics – MBLs are active with Fe(II)

Initial results using electrospray ionisation mass spectrometry under non-denaturing conditions showed that the apo-form of BcII (measured: 24,951 Da, calculated: 24,961 Da) could be reconstituted by addition of Zn(II) or Fe(II), with mass shifts of + 130 and + 108, corresponding to the di-Zn(II) (measured: 25,081 Da, calculated: 25,091 Da) or di-Fe(II) (measured: 25,059 Da, calculated: 25,069 Da) enzymes respectively. The di-metallated enzymes were observed as the major species by mass spectrometry after addition of ≥ 2.5 equivalents of metal ion.

In order to investigate whether these reconstituted enzymes exhibited β-lactamase activity, we performed kinetic studies with both the model MBL, BcII, and the clinically relevant MBL, VIM-2 [10], [48]. Steady-state kinetic studies were performed using both the reporter substrate nitrocefin and meropenem, a clinically used carbapenem antibiotic [49], as substrates, substituting the apo-enzyme with either Zn(II) or Fe(II) as the metal cofactor. Anaerobic conditions, employing a glovebox (< 10 ppm O_2_) [50], [51], were used for the analysis with Fe(II) in order to prevent its oxidation (Table 1 & Table S1 and Figs. S1 & S2). Both apo-BcII and apo-VIM-2 displayed β-lactamase activity when reconstituted not only with Zn(II)SO_4_·7H_2_O, as expected, but also with (NH_4_)_2_Fe(II)(SO_4_)_2_·6H_2_O. Interestingly, and in contrast to literature reports [23], [24], in which no indication of attempts to control O_2_ availability were reported, both Fe(II)-substituted BcII (di-Fe(II) BcII) and VIM-2 (di-Fe(II) VIM-2) were able to efficiently catalyse the hydrolysis of both the nitrocefin and meropenem under conditions of low O_2_. The β-lactamase activity of di-Fe(II) BcII is comparable to that of the di-Zn(II) enzyme, in terms of *k*_*cat*_/*K*_*m*_ values, with both substrates. However, in these assays VIM-2 shows around a 10-fold lower efficiency with both substrates when using the di-Fe(II) enzyme compared to the di-Zn(II) enzyme.

### 3.2 Stopped-flow kinetics – Fe(II) alters the mechanism of β-lactam hydrolysis

**Fig. 2:**
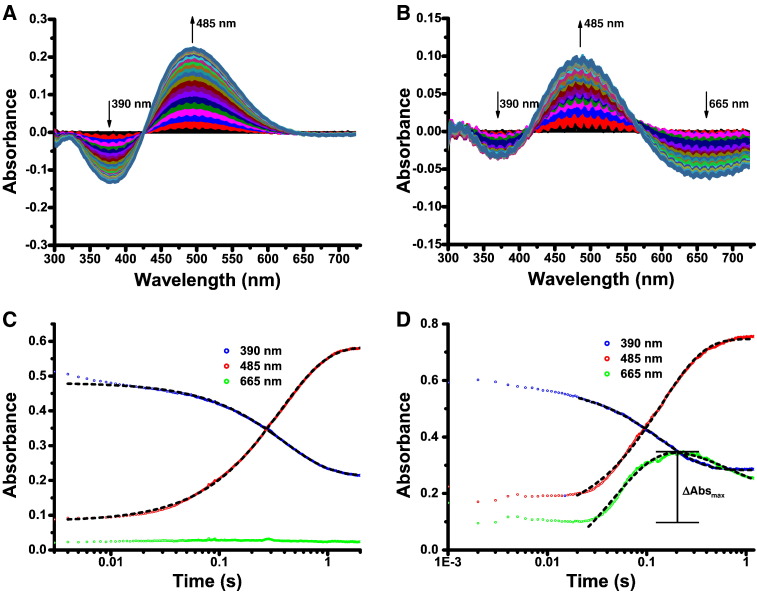
Stopped-flow kinetics of BcII-catalysed nitrocefin hydrolysis. A & B. Spectral changes during the reaction of 50 μM di-Zn(II) BcII, or di-Fe(II) BcII, respectively, with 50 μM nitrocefin in a 1:1 ratio at pH 7.5 and 5 °C. Difference spectra of absorbance wavelengths 300–750 nm from 0.3–1.2 s using absorbance at 0.3 s as a baseline. Arrows indicate growth or decay of peaks. C & D. Time course of the reaction of 50 μM di-Zn(II) BcII, or di-Fe(II) BcII, respectively, with 50 μM nitrocefin in a 1:1 ratio of enzyme to substrate at pH 7.5 and 5 °C. Absorbance traces at 390, 485 and 665 nm. Dashed lines indicate fitting curve traces.

We then employed stopped-flow absorption spectroscopy kinetics to monitor the reaction of di-Zn(II) and di-Fe(II) BcII and VIM-2 in a 1:1 ratio with nitrocefin under the same conditions. Nitrocefin absorbs at 390 nm, with the sometimes observed intermediate derived from it at 665 nm, and the hydrolysed nitrocefin product absorbs at 485 nm (Fig. 1) [52], [53], [54], [55]. Since pH-dependent variation in activity has previously been observed with BcII, we investigated the BcII- and VIM-2-catalysed hydrolysis of nitrocefin at different pH values, i.e. 5.5, 6.5 and 7.5 [17]. As previously reported with di-Zn(II) BcII [56], spectral changes corresponding to substrate depletion (390 nm) and product accumulation (485 nm) were observed with both di-Zn(II) and di-Fe(II) BcII and VIM-2 enzymes (Fig. 2 & Figs. S3–9) [56]. With di-Fe(II) BcII, although no clear feature was observed around 665 nm in the normal absorbance spectra, difference spectra, using the absorbance spectrum at an intermediate time point as a baseline, revealed a decaying feature with a λ_max_ of 665 nm (Fig. 2 & Fig. S3). Note that difference spectra were employed to identify the λ_max_ of the feature and indicate that the feature corresponds to an independent species. In contrast, no such species was observed in the reaction catalysed by di-Zn(II) BcII, in agreement with previous studies (Fig. 2) [56]. A clear feature at 665 nm was observed with di-Zn(II) VIM-2 and nitrocefin, while there are only minor changes in absorbance at this wavelength with di-Fe(II) VIM-2 (Figs. S7–9). Thus the observation of a visible reaction intermediate absorbing at 665 nm depends on the particular metal-enzyme combination employed. No hydrolysis of the substrate was observed in metal salt supplemented buffers over the course of the enzyme-catalysed reaction (Fig. S10).

Kinetic traces for the di-Zn(II) BcII and di-Zn(II) VIM-2 reaction time courses were successfully fitted by a single exponential function at all pH values (Fig. 2, Figs. S4–5 and Figs. S7–9). We were able to successfully fit the changes in absorbance at 665 nm with both di-Fe(II) BcII and di-Zn(II) VIM-2 at pH 7.5 and 6.5, and with di-Zn(II) VIM-2 at pH 5.5, by a double exponential function. However, we were unable to successfully fit the 665 nm absorbance trace for di-Fe(II) BcII at pH 5.5 (Fig. S4) or the 665 nm absorbance traces in general for di-Fe(II) VIM-2 (Figs. S7–9) in the same manner; this is likely due to the shallow gradients of the traces on account of small changes observed in the absorbance at 665 nm.

The fitting constants obtained for the BcII and VIM-2 catalysed reactions are shown in Table 2 & Table S2, respectively. In the case of nitrocefin hydrolysis catalysed by di-Zn(II) BcII, the fitting constants for substrate decay and product accumulation are consistent with a mechanism in which a reaction intermediate is not observed. In contrast, the sometimes observed absorbance changes at 665 nm suggest more than one possible mechanism. This proposal is supported by the differing effects of pH on the di-Zn(II) and di-Fe(II) enzymes; the rate of substrate depletion by di-Zn(II) BcII decreases at pH 7.5 compared to pH 5.5 (2.55 s^− 1^ vs 4.16 s^− 1^), whereas the reverse is seen with di-Fe(II) BcII (7.25 s^− 1^ at pH 7.5 vs 3.88 s^− 1^ at pH 5.5) (Table 2). In contrast, di-Zn(II) VIM-2 is more active at higher pH (substrate decay of 320 s^− 1^ at pH 7.5 vs 190 s^− 1^ at pH 5.5) while di-Fe(II) VIM-2 shows the opposite effect, with a very low rate of substrate depletion at both pH 7.5 and 6.5 (0.15 s^− 1^ and 0.24 s^− 1^, respectively) compared to that at pH 5.5 (35 s^− 1^) (Table S2).

Spectral analysis of the hydrolysis of nitrocefin by di-Zn(II) BcII shows no evidence for an intermediate [56]. In contrast, di-Fe(II) BcII shows a feature at 665 nm, potentially corresponding to an anionic intermediate (Fig. 2). The observation of an intermediate at 665 nm with di-Fe(II) BcII strongly correlates with increasing pH, with a larger change in absorbance amplitude at pH 7.5 (ΔAbs_max_ at pH 7.5 is 0.25 compared to ΔAbs_max_ at pH 6.5 and 5.5 of 0.15 and 0.05, respectively, Fig. 2 & Figs. S4–5). The decay of the 665 nm feature with di-Fe(II) BcII is slower than product accumulation, suggesting that di-Fe(II) BcII might employ a branched reaction pathway as an alternative to the typically proposed linear pathway proceeding through an anionic intermediate (Fig. 4). Data fitting using the Kintek Explorer package [57] was unable to distinguish between linear and branched pathways (Fig. S11), but in both fittings the decay of the intermediate species was identified as the rate-limiting step (*k*_3_ and *k*_4_ in Tables S4 and S5, respectively). Although intermediate decay is apparently rate limiting, a negligible amount of intermediate is seen at pH 5.5. This may be explained either by a branched pathway, in which the branch with no intermediate is favoured by lower pH, or by a linear pathway in which the rate of decay of the intermediate rises with a decrease in pH thus preventing an observable accumulation of intermediate.

Fitting values for di-Zn(II) VIM-2, where an intermediate at 665 nm was seen, give good support to an on pathway intermediate as observed in studies with a similar substrate, chromacef, which is similar to nitrocefin [21]. The rate of formation of this intermediate is increased at higher pH and there is a corresponding increase in its rate of decay. There was no appreciable change in absorbance at 665 nm with di-Fe(II) VIM-2 at all pH values. Thus, nitrocefin hydrolysis by di-Zn(II) VIM-2 proceeds through an anionic intermediate which is no longer apparent upon substitution with Fe(II) (Fig. 4).

Collectively, the results reveal that the mechanism of hydrolysis of nitrocefin by an MBL may change on metal substitution, as demonstrated by the observation, or not, of an intermediate species and by the introduction of a branched reaction pathway (Fig. 4). Differing rates of reaction with different enzyme, metal and pH combinations indicate that the optimal pH for β-lactam hydrolysis is both metal- and enzyme-dependent, a result consistent with literature reports on MBLs [17], [58], [59].

### 3.3 Crystallography – Di-Zn(II) BcII and di-Fe(II) BcII are structurally similar

Having established the activity of di-Fe(II) BcII, we investigated differences in the active site geometry when compared to that of the native di-Zn(II) enzyme using crystallography. We successfully crystallised the Fe(II)-substituted enzyme under low oxygen conditions [60]. Additionally, an apo-preparation of BcII was also crystallised and the resultant structure revealed a lack of metal, validating our metal-free preparations. (Structural data not shown, since our results were identical to a reported apo-BcII structure, PDB accession code: 3I0V [61]). A crystal structure of di-Fe(II) BcII was determined to 1.1 Å resolution. Initial phases were obtained by molecular replacement using a reported structure for BcII complexed with two zinc ions, PDB accession code: 4C09, as a search model [16]. The 4C09 structure was used in structural comparisons since the data are of a comparable quality and resolution to the di-Fe(II) data and were obtained under similar crystallisation conditions. Numerical information on data collection and refinement can be found in Table S3.

**Fig. 3:**
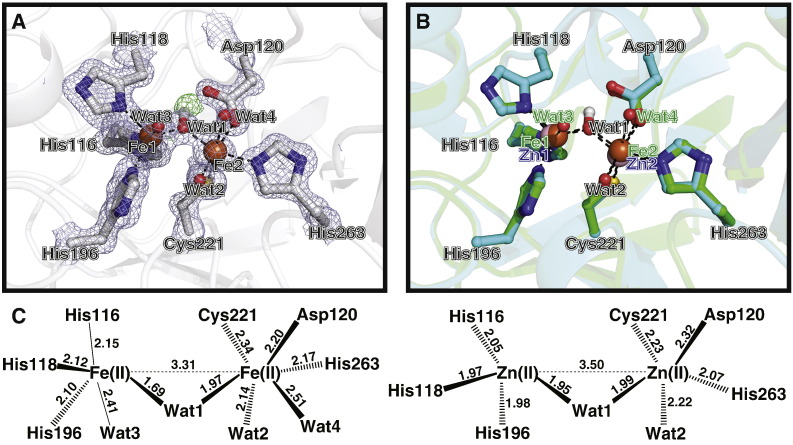
Comparison of the active sites of di-Fe(II) BcII (1.1 Å resolution) and di-Zn(II) BcII (1.2 Å resolution). A. Active site of di-Fe(II)-BcII structure shown with representative electron density (3.0 σ mFo-DFc OMIT, blue mesh). The position of the modelled hydrogen atom associated with the bridging hydroxide ion and its representative density are shown (3.0 σ mFo-DFc OMIT, green mesh). B. Comparison of di-Fe(II) (green) and di-Zn(II) BcII (cyan, PDB Code: 4C09). The amino acid residues occupy very similar positions in both structures but there are clear shifts in the positions of the metal ions. In addition each of the two ferrous ions binds an additional water molecule. BBL numbering is used throughout. C. Views of the active sites of the di-Fe(II) BcII complex (1.1 Å resolution) and di-Zn(II) BcII (PDB code: 4C09, 1.2 Å resolution). Numbers shown represent distances in Å.

The overall structure of di-Fe(II) BcII is almost identical to that of the di-Zn(II) enzyme with an RMSD of 0.084 Å over backbone atoms. Analysis of the active site of the di-Fe(II) structure clearly reveals the binding of two metal centres and that the overall active site structure is retained, notably including retention of a water molecule bridging the two metal centres as described in almost all reported di-Zn(II) MBL structures (Fig. 3). The refined metal occupancies were 1.00 for Fe1 and 0.60 for Fe2. Some additional electron density seen in the mFo-DFc map of the di-Fe(II) structure during refinement led us to model a fraction of Cys221 as a doubly-oxidised sulfur (sulfinic acid) residue (20% population), as precedented by previous work with BcII [61], alongside the cysteine residue which formed the major population (80%) (Fig. S13).

The positions of the metal ligands in di-Fe(II) BcII are almost identical to those of the di-Zn(II) enzyme (PDB accession code: 4C09) with a model RMSD of 0.068 Å over the active site side chain atoms. However, there are significant changes in the relative positions of the metal centres when compared to the di-Zn(II) structure. In Site 1 the iron is shifted by 0.2 Å and in Site 2 it is shifted by 0.4 Å, relative to the Zn positions in the 4C09 model. In addition, in the di-Fe(II) structure both of the ferrous ions have an additional water ligand when compared to the di-Zn(II) structure making Site 1 pentacoordinate with a distorted trigonal bipyramidal geometry; Site 2 is hexacoordinate with a near perfect octahedral coordination sphere. Interestingly, the metal-metal distance in the di-Fe(II) BcII structure is 3.3 Å compared to 3.7 Å as observed for the di-Zn(II) structure 4C09. Further, the distance between the first metal centre and the bridging water molecule shortens from 1.95 Å in di-Zn(II) BcII to 1.69 Å in di-Fe(II) BcII (Fig. 3C). Additional mFo-DFc density seen between the site of the bridging water and Asp120 led us to model the bridging water ligand, Wat1, as a hydroxide with its hydrogen positioned between its oxygen and the side chain oxygen of Asp120, as shown in Fig. 3A.

### 3.4 Inhibition – differently metallated MBLs can respond differently to inhibitors

MBL inhibition is a current target for medicinal chemistry [62], [63]. Given the observed differences in metal binding geometries between the di-Fe(II) and di-Zn(II) form of BcII and in the kinetic properties of di-Fe(II) BcII and VIM-2 compared to their di-Zn(II) enzymes, we then investigated whether the two metal-substituted BcII enzymes might respond differently to known MBL inhibitors (Table 3 and Fig. S12). We were particularly interested to see if the two enzyme forms responded differently to thiol-based MBL inhibitors, since the sulfur often bridges the two metal centres as an analogue of the bridging water during inhibition [16], [64]. The di-Zn(II) and di-Fe(II) enzymes were pre-incubated for 5 min with inhibitors before the addition of nitrocefin as a reporter substrate. We used EDTA, to investigate the ability of BcII to bind the two metals, and two sulfur-based inhibitors – thiomandelic acid [65], which has been found to be a broad spectrum inhibitor of MBLs, and the thioenolate ML302F, which has been shown recently to be a potent inhibitor of B1 MBLs [66].

Under our experimental conditions, the EDTA IC_50_ value for di-Zn(II) BcII was similar to that seen for the di-Fe(II) enzyme (7.5 and 4.7 mM respectively). However, given that the affinity of EDTA for Zn(II) is higher than that for Fe(II) [67], this data suggests that the MBL active site has a greater affinity for Zn(II) ions than for Fe(II) ions, consistent with work characterising Zn(II) ions as, at least, the preferred endogenous MBL metal [68]. Interestingly, the IC_50_ values obtained for thiomandelic acid were very close for the differently metal-substituted forms of BcII. This may be in contrast to what is expected given the high affinity of Zn(II) for sulfur ligands [68]. However, around a 10-fold difference in IC_50_ was obtained with ML302F, an inhibitor shown to exhibit a bidentate binding mode in which a sulfur atom bridges the two metal centres while the adjacent carboxylate binds to the metal in site 2 [66]. This difference may arise, at least in part, as a consequence of the observed shortened intermetal distance and more tightly bound water in the di-Fe(II) BcII structure (Fig. 3C); the alternate position and geometry of the bridging water, Wat1, may make it more difficult for some sulfur ligands to bind in an analogous manner, for steric reasons.

## 4 Discussion

The results reveal that the class B1 MBLs are able to bind Fe(II) and, in contrast to previous reports [11], [22], [23], [24], are active when their Zn(II) ions are replaced by Fe(II) ions under low oxygen conditions, with the enzymes being able to hydrolyse both the chromogenic substrate nitrocefin and the clinically employed carbapenem antibiotic meropenem. Despite differences in the active site metal binding chemistry, in the case of BcII, differences in the overall catalytic efficiencies of di-Zn(II) and di-Fe(II) enzymes are small, whereas di-Fe(II) VIM-2 exhibits around a 10-fold drop in catalytic efficiency with both of the tested substrates. The differences between our results and previous reports of a lack of activity for MBLs with iron likely result from oxidation of the ferrous iron.

**Fig. 4:**
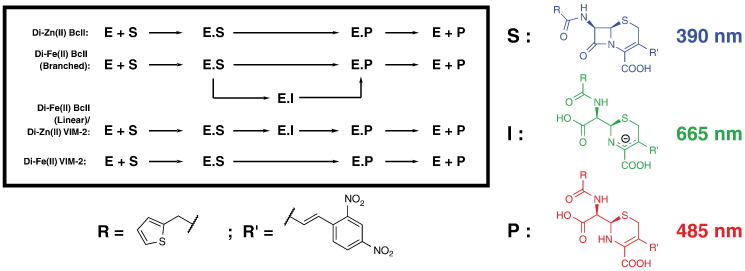
Proposed possible reaction schemes for differently metal-substituted BcII and VIM-2. Di-Zn(II) BcII manifests no observable intermediate, while the hydrolysis of nitrocefin by di-Fe(II) BcII is best described by a branched mechanism. Di-Zn(II) VIM-2 manifests an on pathway intermediate that is not observed with di-Fe(II) VIM-2. The proposed corresponding species, S, I and P, and their absorbance wavelengths, are also shown.

Stopped-flow studies of reconstituted di-Fe(II) BcII with nitrocefin revealed an additional absorbance feature with a λ_max_ of 665 nm. In some cases, previous work with nitrocefin, and the related substrate chromacef, has identified an intermediate, absorbing at 665 nm, corresponding to the deprotonated delocalised anion with MBLs including VIM-2, IMP-1, New Delhi metallo-β-lactamase 1 (NDM-1) and L1 [21], [52], [53], [54], [69], [70], [71]. Such a feature was not visible in the di-Zn(II) BcII reaction spectrum, as has been previously reported [56], suggesting that di-Fe(II) BcII may employ a different mechanism for β-lactam hydrolysis (Fig. 4). In contrast the on-pathway intermediate seen in nitrocefin hydrolysis by di-Zn(II) VIM-2 is no longer apparent on substitution with Fe(II).

The crystallographic results reveal that the overall geometry of the BcII active site is retained on metal substitution with Fe(II), including the presence of a bridging water molecule (Fig. 3). However, there are changes in the positions of the metal centres resulting in different coordination distances and angles between both the metal ligands and the bridging water. Although it would be premature to propose any mechanistic consequences of the shifted metal binding sites, the results at least raise the possibility that changes in metal position may occur in catalysis and indeed inhibition; emerging evidence with non-haem Fe(II)-dependent oxygenases suggests that changes in metal position in some enzymes may have been underestimated to date [72], [73], [74], [75]. In the di-Fe(II) BcII structure, the presence of extra mFo-DFc density beside the bridging oxygen lead us to model the bridging species as a hydroxide ion with its hydrogen projecting towards Asp120. In addition to the essential metal ligation, Asp120 has been proposed to play additional roles in MBL catalysis including orientating a zinc-bound bridging water to protonate the anionic intermediate [76]. Our results suggest that Asp120 may also play a role in positioning the bridging hydroxide to attack the β-lactam carbonyl in the enzyme-substrate complex.

It has previously been reported that a number of MBL enzymes can co-purify with iron ions in the active site [26], [35], [36], [37]. This is not unsurprising given the promiscuous ability of these enzymes to bind and exhibit catalysis with a number of transition metal ions. Indeed there are members of the MBL superfamily that bind Fe(II) in their native forms to carry out their catalytic functions [31], [32], [33], [35]. Iron is a relatively bioavailable metal, although not necessarily in an available reduced ferrous form, giving rise to the possibility that Fe(II)-substituted MBLs may constitute a proportion of the active enzyme population, especially under conditions of relatively low Zn(II) or high Fe(II) availability [77], [78]. This possibility is supported by the finding that Pce, an Fe(II)-dependent MBL-fold hydrolase is an extracellularly located protein [35], [79]. We believe that the knowledge that there already exists a bacterial di-Fe(II)-binding MBL fold enzyme that exhibits extracellular activity lends credence to our proposal that di-Fe(II)-bound true MBLs might constitute a biologically relevant population.

The inhibitor studies reveal that in some, but not all cases, it is possible for the same inhibitor to behave differently with di-Zn(II) and di-Fe(II) BcII (Table 3), a result consistent with the altered active site metallo-chemistry between the di-Zn(II) and di-Fe(II) forms (Fig. 3). The potent inhibitor ML302F exhibited a higher IC_50_ with di-Fe(II) BcII than di-Zn(II) BcII [66]. Thus, it may be prudent to test MBL inhibitors that are in clinical development against both Zn(II)- and Fe(II)-bound MBLs, in order to identify inhibitors potent against both metallated forms. Further, given the known promiscuity of the MBL fold, it is possible that substitution of Zn(II) for other metals may alter catalytic properties in a manner relevant to the development of resistance. In this regard it is of interest that a recent report has highlighted the effects of different active site metals on native compared to promiscuous reactions as catalysed by MBL fold proteins [24].

## Declarations of interest

The authors declare no conflict of interest.

## Funding information

We thank the Biotechnology and Biological Sciences Research Council (grant number BB/J014427/1) and the Medical Research Council/Canadian grant (G1100135) for funding our work.

## Accession number

Coordinates and structure factors have been deposited in the Protein Data Bank with accession number 5FQA.

## Figures and Tables

**Figure:**
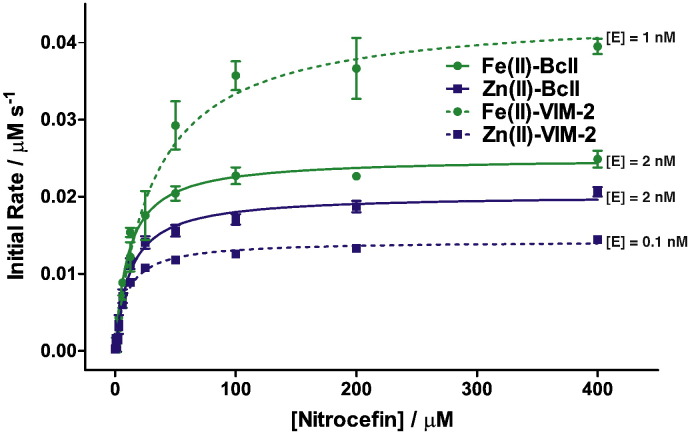
Supplementary figure

**Table 1:** Kinetic constants for the reaction of metal-substituted BcII and VIM-2 with nitrocefin. Data were fitted using GraphPad Prism 5.

Enzyme	Metal	[E] (nM)	*k*_*cat*_ (s^− 1^)	*K*_*m*_ (μM)	*k*_*cat*_/*K*_*m*_ (μM^− 1^ s^− 1^)	Literature value *k*_*cat*_/*K*_*m*_ (μM^− 1^ s^− 1^)
BcII	Zn(II)	2	10.1 ± 0.4	12 ± 2	0.82	1.8^[40]^
BcII	Fe(II)	2	12.5 ± 0.4	11 ± 2	1.2	–
VIM-2	Zn(II)	0.1	143 ± 3	9 ± 1	16	31.2^[40]^
VIM-2	Fe(II)	1	44 ± 2	32 ± 5	1.4	–

**Table 2:** Analysis of the reaction of di-Zn(II) and di-Fe(II) BcII with nitrocefin in a 1:1 ratio. Fitting constants are obtained from analysis of reaction time courses seen in Fig. 1 & Figs. S4–5. Substrate decay, product accumulation and intermediate accumulation and decay correspond to absorbance features at 390, 485 and 665 nm, respectively.

Metal	pH	Substrate decay (s^− 1^)	Intermediate accumulation (s^− 1^)	Intermediate decay (s^− 1^)	Product accumulation (s^− 1^)
Zn(II)	5.5	4.16 ± 0.01	–	–	4.13 ± 0.01
Zn(II)	6.5	3.24 ± 0.01	–	–	3.29 ± 0.01
Zn(II)	7.5	2.55 ± 0.01	–	–	2.75 ± 0.01
Fe(II)	5.5	3.88 ± 0.01	Not fitted	Not fitted	5.78 ± 0.01
Fe(II)	6.5	5.25 ± 0.01	18.31 ± 0.01	1.2 ± 0.1	4.27 ± 0.01
Fe(II)	7.5	7.25 ± 0.01	20.32 ± 0.01	1.4 ± 0.1	7.20 ± 0.01

**Table 3:**
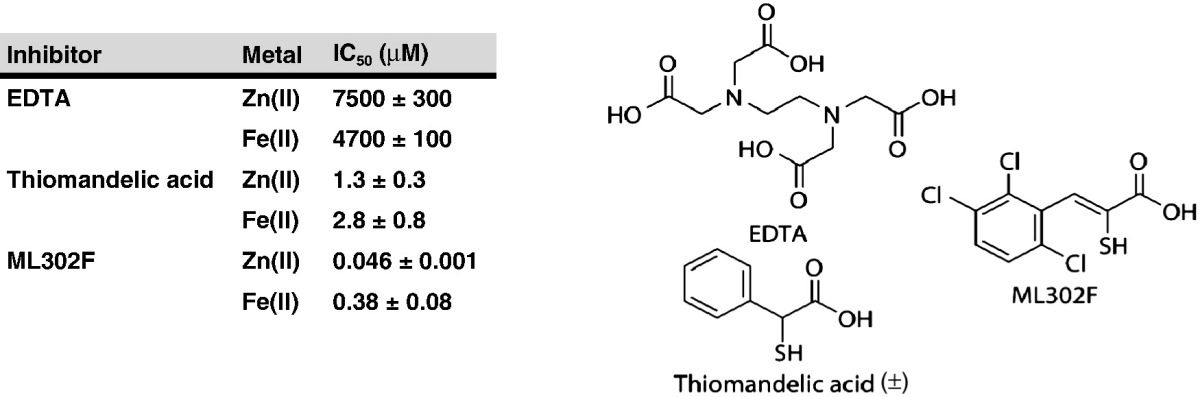
IC_50_ values for inhibitors of metal-substituted BcII, obtained from fitting of residual activity plots using GraphPad Prism. The chemical structures of the inhibitors employed are provided.

## Appendix A Supplementary data

Supplementary material 1 Image 2

Supplementary material 2 Image 3

## References

[1] Versporten A., Bolokhovets G., Ghazaryan L., Abilova V., Pyshnik G., Spasojevic T., Korinteli I., Raka L., Kambaralieva B., Cizmovic L., Carp A., Radonjic V., Maqsudova N., Celik H.D., Payerl-Pal M., Pedersen H.B., Sautenkova N., Goossens H. (2014). Lancet Infect. Dis..

[2] Blair J.M.A., Webber M.A., Baylay A.J., Ogbolu D.O., Piddock L.J.V. (2015). Nat. Rev. Microbiol..

[3] Majiduddin F.K., Materon I.C., Palzkill T.G. (2002). Int. J. Med. Microbiol..

[4] Bush K. (2010). Crit. Care.

[5] Palzkill T. (2013). Ann. N. Y. Acad. Sci..

[6] Bush K., Jacoby G.A. (2010). Antimicrob. Agents Chemother..

[7] Ambler R.P. (1980). Philos. Trans. R. Soc. B.

[8] Queenan A.M., Bush K. (2007). Clin. Microbiol. Rev..

[9] Walsh T.R. (2010). Int. J. Antimicrob. Agents.

[10] Carfi A., Pares S., Duée E., Galleni M., Duez C., Frère J.M., Dideberg O. (1995). EMBO J..

[11] Morán-Barrio J., González J.M., Lisa M.N., Costello A.L., Peraro M.D., Carloni P., Bennett B., Tierney D.L., Limansky A.S., Viale A.M., Vila A.J. (2007). J. Biol. Chem..

[12] Hernandez Valladares M., Felici A., Weber G., Adolph H.W., Zeppezauer M., Rossolini G.M., Amicosante G., Frère J.-M., Galleni M. (1997). Biochemistry.

[13] Bebrone C. (2007). Biochem. Pharmacol..

[14] Garau G., García-Sáez I., Bebrone C., Anne C., Mercuri P., Galleni M., Frère J.-M., Dideberg O. (2004). Antimicrob. Agents Chemother..

[15] Fabiane S.M., Sohi M.K., Wan T., Payne D.J., Bateson J.H., Mitchell T., Sutton B.J. (1998). Biochemistry.

[16] Brem J., van Berkel S.S., Zollman D., Lee S.Y., Gileadi O., McHugh P.J., Walsh T.R., McDonough M.A., Schofield C.J. (2016). Antimicrob. Agents Chemother..

[17] Badarau A., Page M.I. (2006). Biochemistry.

[18] Hu Z., Spadafora L.J., Hajdin C.E., Bennett B., Crowder M.W. (2009). Biochemistry.

[19] Badarau A., Damblon C., Page M.I. (2007). Biochem. J..

[20] Tioni M.F., Llarrull L.I., Poeylaut-Palena A.A., Martí M.A., Saggu M., Periyannan G.R., Mata E.G., Bennett B., Murgida D.H., Vila A.J. (2008). J. Am. Chem. Soc..

[21] Aitha M., Marts A.R., Bergstrom A., Møller A.J., Moritz L., Turner L., Nix J.C., Bonomo R.A., Page R.C., Tierney D.L., Crowder M.W. (2014). Biochemistry.

[22] Hu Z., Periyannan G., Bennett B., Crowder M.W. (2008). J. Am. Chem. Soc..

[23] Hu Z., Gunasekera T.S., Spadafora L., Bennett B., Crowder M.W. (2008). Biochemistry.

[24] Baier F., Chen J., Solomonson M., Strynadka N.C.J., Tokuriki N. (2015). ACS Chem. Biol..

[25] Liu D., Lepore B.W., Petsko G.A., Thomas P.W., Stone E.M., Fast W., Ringe D. (2005). Proc. Natl. Acad. Sci. U. S. A..

[26] Crowder M.W., Maiti M.K., Banovic L., Makaroff C.A. (1997). FEBS Lett..

[27] Dong Y.-J., Bartlam M., Sun L., Zhou Y.-F., Zhang Z.-P., Zhang C.-G., Rao Z., Zhang X.-E. (2005). J. Mol. Biol..

[28] Vogel A., Schilling O., Niecke M., Bettmer J., Meyer-Klaucke W. (2002). J. Biol. Chem..

[29] Mandel C.R., Kaneko S., Zhang H., Gebauer D., Vethantham V., Manley J.L., Tong L. (2006). Nature.

[30] Cattell E., Sengerová B., McHugh P.J. (2010). Environ. Mol. Mutagen..

[31] Frazao C., Silva G., Gomes C.M., Matias P., Coelho R., Sieker L., Macedo S., Liu M.Y., Oliveira S., Teixeira M., Xavier A.V., Rodrigues-Pousada C., Carrondo M.A., Le Gall J. (2000). Nat. Struct. Mol. Biol..

[32] Silaghi-Dumitrescu R., Kurtz D.M., Ljungdahl L.G., Lanzilotta W.N. (2005). Biochemistry.

[33] Pettinati I., Brem J., McDonough M.A., Schofield C.J. (2015). Hum. Mol. Genet..

[34] McCoy J.G., Bingman C.A., Bitto E., Holdorf M.M., Makaroff C.A., Phillips G.N. (2006). Acta Crystallogr. Sect. D: Biol. Crystallogr..

[35] Garau G., Lemaire D., Vernet T., Dideberg O., Di Guilmi A.M. (2005). J. Biol. Chem..

[36] Cameron A.D., Ridderström M., Olin B., Mannervik B. (1999). Structure.

[37] Thomas P.W., Zheng M., Wu S., Guo H., Liu D., Xu D., Fast W. (2011). Biochemistry.

[38] Skaar E.P. (2010). PLoS Pathog..

[39] de Seny D., Prosperi-Meys C., Bebrone C., Rossolini G.M., Page M.I., Noel P., Frère J.-M., Galleni M. (2002). Biochem. J..

[40] van Berkel S.S., Brem J., Rydzik A.M., Salimraj R., Cain R., Verma A., Owens R.J., Fishwick C.W.G., Spencer J., Schofield C.J. (2013). J. Med. Chem..

[41] OriginPro 8.5.1, Northampton, Massachusetts, USA

[42] Makena A., van Berkel S.S., Lejeune C., Owens R.J., Verma A., Salimraj R., Spencer J., Brem J., Schofield C.J. (2013). ChemMedChem.

[43] GraphPadPrism 5.04, San Diego, California, USA

[44] Battye T.G.G., Kontogiannis L., Johnson O., Powell H.R., Leslie A.G.W. (2011). Acta Crystallogr. Sect. D: Biol. Crystallogr..

[45] Evans P.R., Murshudov G.N. (2013). Acta Crystallogr. Sect. D: Biol. Crystallogr..

[46] Adams P.D., Grosse-Kunstleve R.W., Hung L.-W., Ioerger T.R., McCoy A.J., Moriarty N.W., Read R.J., Sacchettini J.C., Sauter N.K., Terwilliger T.C. (2002). Acta Crystallogr. Sect. D: Biol. Crystallogr..

[47] Emsley P., Lohkamp B., Scott W.G., Cowtan K. (2010). Acta Crystallogr. Sect. D: Biol. Crystallogr..

[48] Garcia-Saez I., Docquier J.D., Rossolini G.M., Dideberg O. (2008). J. Mol. Biol..

[49] Edwards J.R., Turner P.J., Wannop C., Withnell E.S., Grindey A.J., Nairn K. (1989). Antimicrob. Agents Chemother..

[50] Zhang Z., Kochan G.T., Ng S.S., Kavanagh K.L., Oppermann U., Schofield C.J., McDonough M.A. (2011). Biochem. Biophys. Res. Commun..

[51] Zhang Z., Ren J.-S., Clifton I.J., Schofield C.J. (2004). Chem. Biol..

[52] McManus-Munoz S., Crowder M.W. (1999). Biochemistry.

[53] Spencer J., Clarke A.R., Walsh T.R. (2001). J. Biol. Chem..

[54] Yang H., Aitha M., Hetrick A.M., Richmond T.K., Tierney D.L., Crowder M.W. (2012). Biochemistry.

[55] O'Callaghan C.H., Morris A., Kirby S.M., Shingler A.H. (1972). Antimicrob. Agents Chemother..

[56] Rasia R.M., Vila A.J. (2003). ARKIVOC.

[57] Johnson K.A., Simpson Z.B., Blom T. (2009). Anal. Biochem..

[58] Ohsuka S., Arakawa Y., Horii T., Ito H., Ohta M. (1995). Antimicrob. Agents Chemother..

[59] Mercuri P.S., Bouillenne F., Boschi L., Lamotte-Brasseur J., Amicosante G., Devreese B., van Beeumen J., Frère J.-M., Rossolini G.M., Galleni M. (2001). Antimicrob. Agents Chemother..

[60] Roach P.L., Clifton I.J., Hensgens C.M.H., Shibata N., Long A.J., Strange R.W., Hasnain S.S., Schofield C.J., Baldwin J.E., Hajdu J. (1996). Eur. J. Biochem..

[61] González J.M., Buschiazzo A., Vila A.J. (2010). Biochemistry.

[62] Drawz S.M., Bonomo R.A. (2010). Clin. Microbiol. Rev..

[63] Fast W., Sutton L.D. (2013). Biochim. Biophys. Acta.

[64] Karsisiotis A.I., Damblon C.F., Roberts G.C.K. (2013). Biochem. J..

[65] Mollard C., Moali C., Papamicael C., Damblon C., Vessilier S., Amicosante G., Schofield C.J., Galleni M., Frère J.-M., Roberts G.C.K. (2001). J. Biol. Chem..

[66] Brem J., van Berkel S.S., Aik W., Rydzik A.M., Avison M.B., Pettinati I., Umland K.-D., Kawamura A., Spencer J., Claridge T.D.W., McDonough M.A., Schofield C.J. (2014). Nat. Chem..

[67] Smith R.M., Martell A.E. (1976). Critical Stability Constants.

[68] González J.M., Meini M.-R., Tomatis P.E., Martín F.J.M., Cricco J.A., Vila A.J. (2012). Nat. Chem. Biol..

[69] Moali C., Anne C., Lamotte-Brasseur J., Groslambert S., Devreese B., Van Beeumen J., Galleni M., Frère J.-M. (2003). Chem. Biol..

[70] Wang Z., Fast W., Benkovic S.J. (1998). J. Am. Chem. Soc..

[71] Oelschlaeger P., Aitha M., Yang H., Kang J.S., Zhang A.L., Liu E.M., Buynak J.D., Crowder M.W. (2015). Antimicrob. Agents Chemother..

[72] Kruidenier L., Chung C.-W., Cheng Z., Liddle J., Che K., Joberty G., Bantscheff M., Bountra C., Bridges A., Diallo H., Eberhard D., Hutchinson S., Jones E., Katso R., Leveridge M., Mander P.K., Mosley J., Ramirez-Molina C., Rowland P., Schofield C.J., Sheppard R.J., Smith J.E., Swales C., Tanner R., Thomas P., Tumber A., Drewes G., Oppermann U., Patel D.J., Lee K., Wilson D.M. (2012). Nature.

[73] Hopkinson R.J., Tumber A., Yapp C., Chowdhury R., Aik W., Che K.H., Li X.S., Kristensen J.B.L., King O.N.F., Chan M.C., Yeoh K.K., Choi H., Walport L.J., Thinnes C.C., Bush J.T., Lejeune C., Rydzik A.M., Rose N.R., Bagg E.A., McDonough M.A., Krojer T.J., Yue W.W., Ng S.S., Olsen L., Brennan P.E., Oppermann U., Muller S., Klose R.J., Ratcliffe P.J., Schofield C.J., Kawamura A. (2013). Chem. Sci..

[74] Oelschlaeger P., Schmid R.D., Pleiss J. (2003). Protein Eng..

[75] Breece R.M., Hu Z., Bennett B., Crowder M.W., Tierney D.L. (2009). J. Am. Chem. Soc..

[76] Meini M.-R., Llarrull L.I., Vila A.J. (2015). FEBS Lett..

[77] Lau C.K.Y., Krewulak K.D., Vogel H.J. (2015). FEMS Microbiol. Rev..

[78] Krewulak K.D., Vogel H.J. (2008). Biochim. Biophys. Acta.

[79] Vollmer W., Tomasz A. (2001). Mol. Microbiol..

