# Supplementary material for: Use of ferrous iron by metallo-β-lactamases
Source: J Inorg Biochem. 2016 Oct;163:185–93. doi: 10.1016/j.jinorgbio.2016.07.013 (PMC5108564; doi:10.1016/j.jinorgbio.2016.07.013)
Supplement: Supplementary file 1 — Supplementary material 1 [file mmc1.docx]

**Use of Ferrous Iron by Metallo-β-Lactamases**

Samuel T. Cahill^a^, Hanna Tarhonskaya^a^, Anna M. Rydzik^b^, Emily Flashman^a^, Michael A. McDonough^a^, Christopher J. Schofield^a†^, and Jürgen Brem^a†^

^a^Chemistry Research Laboratory, Oxford

^b^ Chemistry Research Laboratory, Oxford (Current Address: Ludwig-Maximilians-University, Munich)

^†^Corresponding author (Christopher Schofield email: christopher.schofield@chem.ox.ac.uk, Jürgen Brem email: jurgen.brem@chem.ox.ac.uk)

**Contents**

| **Supplementary Methods** | **Page S3** |
| --- | --- |
| **Supplementary Tables** | **Page S4** |
| **Supplementary Figures** | **Page S6** |
| **References** | **Page S16** |

**Supplementary Methods**

**Preparation of thiomandelic acid**

To a stirred solution of α-bromophenylacetic acid (2 g, 9.3 mmol) in dioxane (10 mL) was added a dropwise solution of potassium thioacetate (3 g, 18.6 mmol) in methanol (5 mL). The resultant mixture was stirred for 12 h at room temperature and then was poured on ice-water (40 mL) and extracted with diethyl ether (3×10 mL). The aqueous phase was acidified with 20% sulphuric acid and extracted with diethyl ether (3×10 mL). The ether extracts were combined, dried over sodium sulphate and concentrated *in vacuo*. The resultant white solid was suspended in concentrated hydrochloric acid (20 mL) and stirred at 50°C for 12 h. The solution was cooled down and poured on ice-water. The resultant precipitate was collected by filtration and dried under vacuum to yield thiomandelic acid as a white solid (0.93 g, 5.6 mmol, 60%).

^1^H NMR (400 MHz, *DMSO-d*_6_) δ ppm 4.68 (s, 1 H), 7.33 - 7.44 (m, 5 H). ^13^C NMR (101 MHz, *DMSO-d*_6_) δ ppm 57.8, 128.9, 129.1, 129.2, 136.1, 171.4. m.p. 120-122°C (lit. 81°C). LRMS (ESI^-^, *m/z*) 167.1 [M-H]. Analytical data are with agreement with published data [[1](#_ENREF_1)].

**Supplementary Tables**

|  |  |  |  |  |  | **Literature Value*** |
| --- | --- | --- | --- | --- | --- | --- |
| **Enzyme** | **Metal** | **[E] (nM)** | ***k_cat_* (s^-1^)** | ***K_m_* (μM)** | ***k_cat_*/*K_m_* (μM^-1^ s^-1^)** | ***k_cat_*/*K_m_* (μM^-1^ s^-1^)** |
| **BcII** | **Zn(II)** | **2** | **89 ± 3** | **220 ± 20** | **0.40** | **-** |
| **BcII** | **Fe(II)** | **2** | **38 ± 2** | **230 ± 30** | **0.17** | **-** |
| **VIM-2** | **Zn(II)** | **5** | **2.4 ± 0.1** | **10 ± 2** | **0.24** | **0.28^[^**[**^2^**](#_ENREF_2)**^]^** |
| **VIM-2** | **Fe(II)** | **30** | **0.5 ± 0.0** | **16 ± 2** | **0.03** | **-** |

**Table S1: Kinetic constants for the reaction of metal-substituted BcII and VIM-2 with meropenem. Data were fitted using GraphPad Prism 5.**

|  |  | **Substrate** | **Intermediate** | **Intermediate** | **Product** |
| --- | --- | --- | --- | --- | --- |
| **Metal** | **pH** | **Decay (s^-1^)** | **Accumulation (s^-1^)** | **Decay (s^-1^)** | **Accumulation (s^-1^)** |
| **Zn(II)** | **5.5** | **190 ± 4** | **220 ± 20** | **120 ± 10** | **93 ± 2** |
| **Zn(II)** | **6.5** | **290 ± 10** | **320 ± 80** | **160 ± 30** | **130 ± 4** |
| **Zn(II)** | **7.5** | **320 ± 30** | **4000 ± 100** | **180 ± 50** | **140 ± 5** |
| **Fe(II)** | **5.5** | **35 ± 1** | **-** | **-** | **23.4 ± 0.5** |
| **Fe(II)** | **6.5** | **0.24 ± 0.01** | **-** | **-** | **0.44 ± 0.01** |
| **Fe(II)** | **7.5** | **0.15 ± 0.01** | **-** | **-** | **0.35 ± 0.01** |

**Table S2: Analysis of the reaction of Zn- and Fe(II)-substituted VIM-2 with nitrocefin in a 1:1 ratio. Fitting constants are obtained from analysis of reaction time courses seen in figures S7-9. Substrate Decay, Product Growth and Intermediate Growth and Decay correspond to absorbance features at 390, 485 and 665 nm, respectively.**

| **Data Set** | **Diferrous-BcII** |
| --- | --- |
| Data Collection |  |
| Source | **Diamond Light Source I02 Beamline** |
| Wavelength (Å) | **0.9795** |
| Resolution Range (Å) | **14.18 – 1.1 (1.139 – 1.1)^a^** |
| Space Group | ***C* 2_1_** |
| Unit Cell Parameters |  |
| *a, b, c* (Å) | **53.1, 61.1, 69.4** |
| α, β, γ (°) | **90.0, 93.1, 90.0** |
| Unique Reflections | **87857 (4468)^a^** |
| Completeness (%) | **97.48 (92.67)^a^** |
| Redundancy | **6.1** |
| R_merge_ | **0.065 (0.10)^a^** |
| <I/σ(I)> | **16.9 (4.9)^a^** |
| Refinement |  |
| R_work_/R_free_ | **0.1173/0.1395** |
| RMSD |  |
| Bonds (Å) | **0.012** |
| Angles (°) | **1.568** |
| Average B-factor for protein atoms (Å^2^) | **16.60** |
| Ramachandran Plot |  |
| Most Favoured Geometry (%) | **96.0** |
| Additionally Allowed (%) | **4.0** |
| Outliers (%) | **0.0** |

**Table S3: Crystallographic data and refinement statistics for di-Fe(II)-BcII. ^a^Values for the highest resolution shell.**

| **Variable** | **Fitted Value** | **% Error** |
| --- | --- | --- |
| **k_1_** | **225000** | **0.73** |
| **k_-1_** | **0.000000403** | **ND** |
| **k_2_** | **82.7** | **3.62** |
| **k_3_** | **7.2** | **0.98** |
| **k_4_** | **100000000** | **fixed** |
|  |  |  |
| **ε_S_** | **6319** | **fixed** |
| **ε_P_** | **12600** | **fixed** |
| **ε_I+S_** | **13820** | **fixed** |
| **ε_I+P_** | **4999** | **fixed** |
| **Offset_S_** | **0.2851** | **fixed** |
| **Offset_P_** | **0.116** | **fixed** |
| **Offset_I_** | **0.01078** | **fixed** |

**Table S4: Kinetic constants obtained from fitting the reaction of 50 μM di-Fe(II) BcII with 50 μM nitrocefin in a 1:1 ratio at pH 7.5 and 5°C using a linear mechanism (see Figure S12). Data fitting was carried out using Kintek Explorer. Fixed values were first allowed to float from initial variables before fixing. ND – Not determined.**

| **Variable** | **Fitted Value** | **% Error** |
| --- | --- | --- |
| **k_1_** | **239000** | **0.84** |
| **k_-1_** | **0.0000001** | **ND** |
| **k_2_** | **46** | **6.59** |
| **k_3_** | **73.4** | **5.79** |
| **k_4_** | **4.18** | **1.9** |
| **k_5_** | **100000000** | **fixed** |
|  |  |  |
| **ε_S_** | **6700** | **fixed** |
| **ε_S+P_** | **13200** | **fixed** |
| **ε_P_** | **10600** | **fixed** |
| **ε_I+S_** | **13500** | **fixed** |
| **ε_I+P_** | **4180** | **fixed** |
| **Offset_S_** | **0.289** | **fixed** |
| **Offset_P_** | **0.0966** | **fixed** |
| **Offset_I_** | **0.0377** | **fixed** |
|  |  |  |

**Table S5: Kinetic constants obtained from fitting the reaction of 50 μM di-Fe(II) BcII with 50 μM nitrocefin in a 1:1 ratio at pH 7.5 and 5°C using a branched mechanism (see Figure S12). Data fitting was carried out using Kintek Explorer. Fixed values were first allowed to float from initial variables before fixing. ND – Not determined.**

**Supplementary Figures**

**
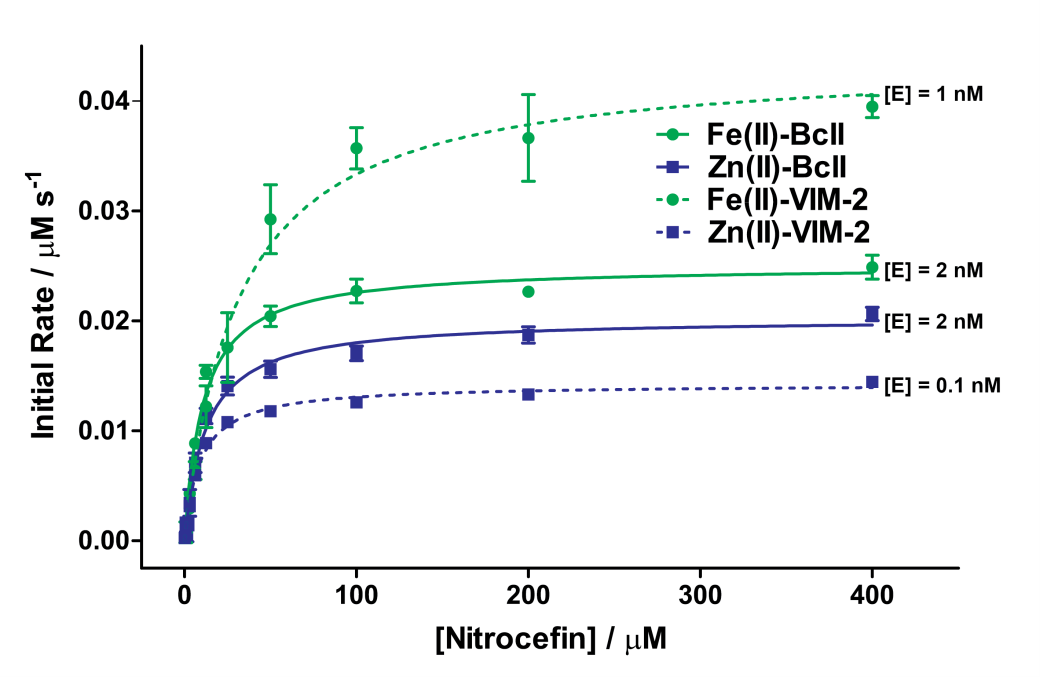
**

**Figure S1: Reaction of metal-substituted BcII and VIM-2 with nitrocefin. The initial rate of the reaction is plotted against substrate concentration. Solid lines indicate the fitted Michaelis-Menten curve for BcII while dashed lines correspond to VIM-2.**

**
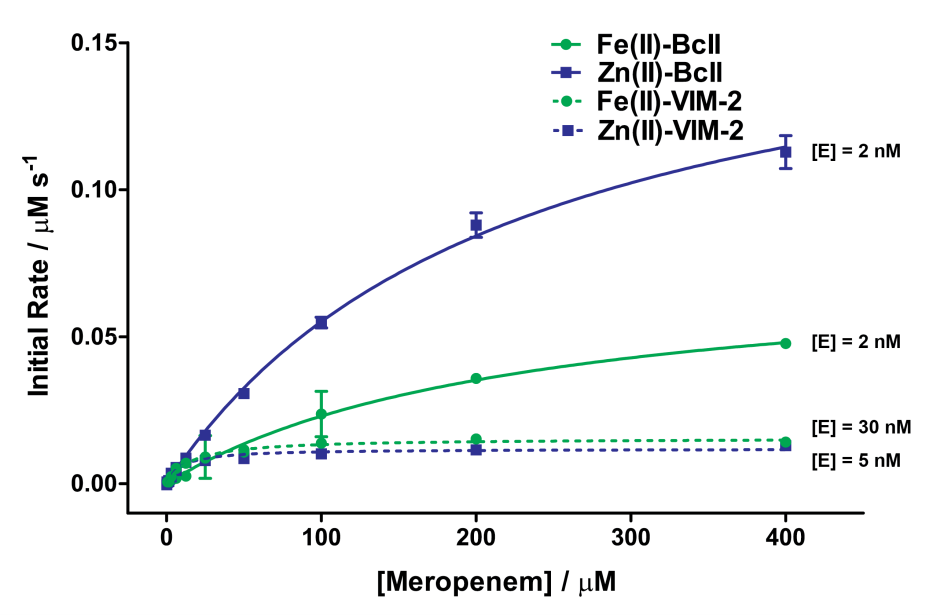
**

**Figure S2: Reaction of metal-substituted BcII and VIM-2 with meropenem. The initial rate of the reaction is plotted against substrate concentration. Solid lines indicate the fitted Michaelis-Menten curve for BcII while dashed lines correspond to VIM-2.**

**
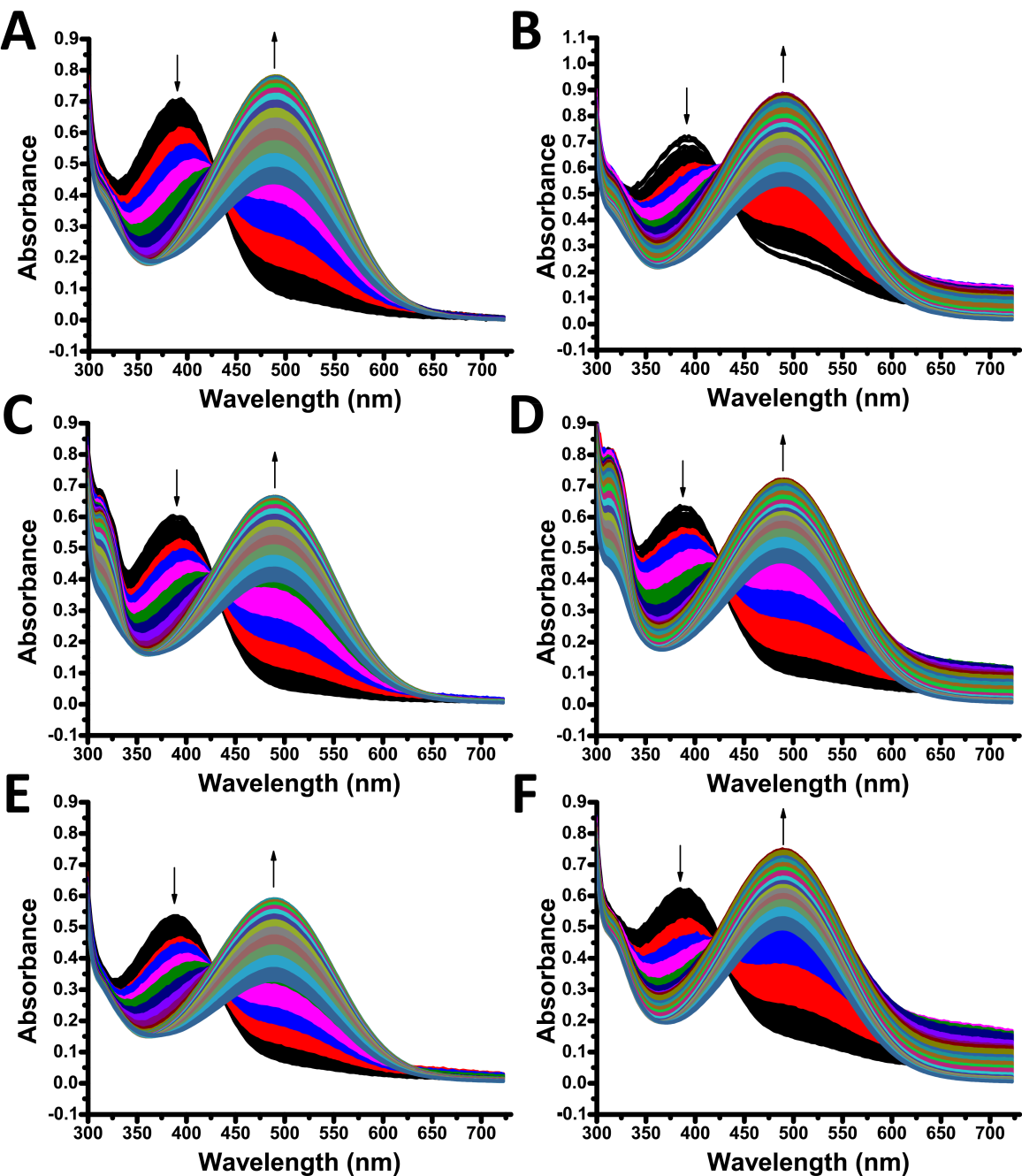
**

**Figure S3: Spectral changes during the reaction of the specified enzyme with nitrocefin in a 1:1 ratio at 5°C. A. Di-Zn(II) BcII, pH 5.5. B. Di-Fe(II) BcII, pH 5.5. C. Di-Zn(II) BcII, pH 6.5. D. Di-Fe(II) BcII, pH 6.5. E. Di-Zn(II) BcII, pH 7.5. F. Di-Fe(II) BcII, pH 7.5.**

**
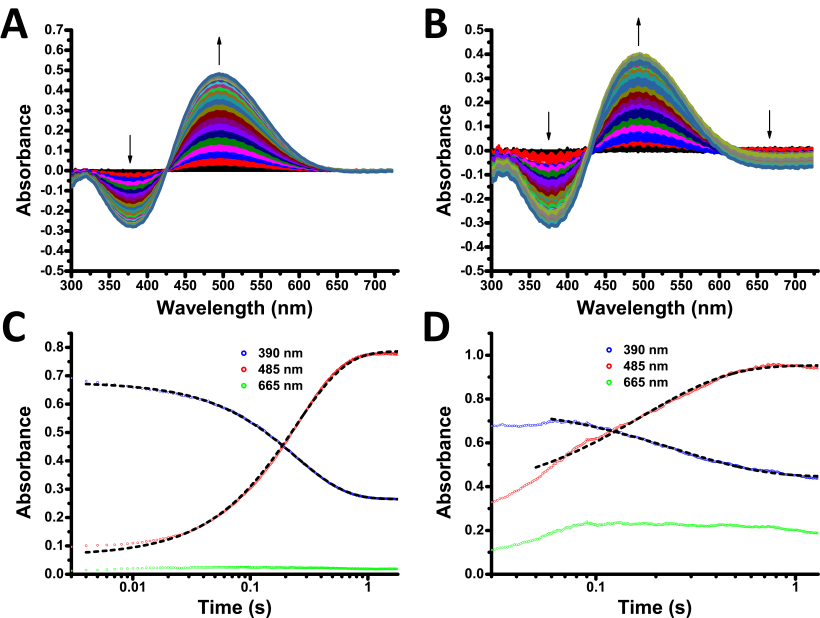
**

**Figure S4: A & B. Spectral changes during the reaction of 50 μM di-Zn(II) BcII, or di-Fe(II) BcII, respectively, with 50 μM nitrocefin in a 1:1 ratio at pH 5.5 and 5°C. Difference spectra of absorbance across wavelengths 300-750 nm from 0.1–1.3 s using absorbance at 0.1 s as a baseline. Arrows indicate growth or decay of peaks. C & D. Time course of the reaction of 50 μM di-Zn(II) BcII, or di-Fe(II) BcII, respectively, with 50 μM nitrocefin in a 1:1 ratio of enzyme to substrate at pH 5.5 and 5°C. Absorbance traces at 390, 485 and 665 nm. Dashed lines indicate fitting curve traces.**

**
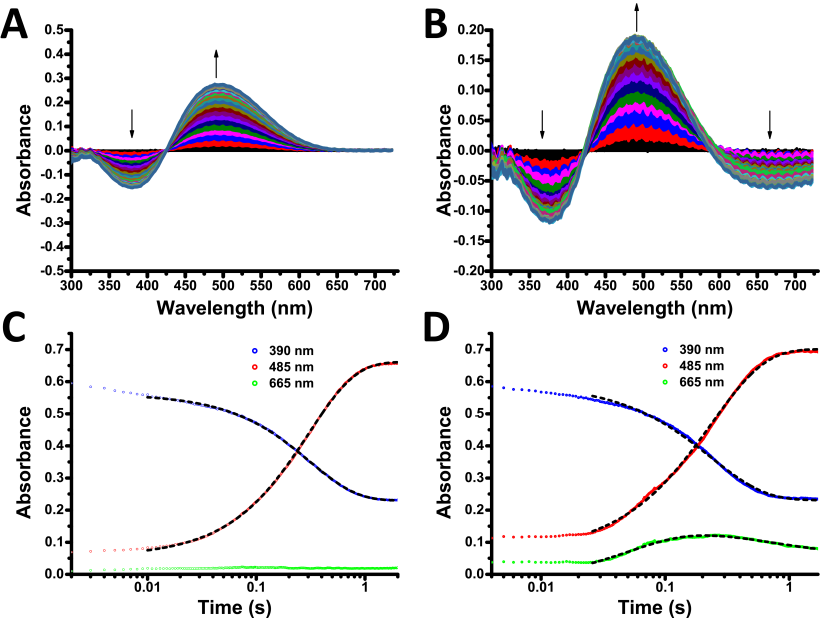
**

**Figure S5: A & B. Spectral changes during the reaction of 50 μM di-Zn(II) BcII, or di-Fe(II) BcII, respectively, with 50 μM nitrocefin in a 1:1 ratio at pH 6.5 and 5°C. Difference spectra of absorbance across wavelengths 300-750 nm from 0.25–1.7 s using absorbance at 0.25 s as a baseline. Arrows indicate growth or decay of peaks. C & D. Time course of the reaction of 50 μM di-Zn(II) BcII, or di-Fe(II) BcII, respectively, with 50 μM nitrocefin in a 1:1 ratio of enzyme to substrate at pH 6.5 and 5°C. Absorbance traces at 390, 485 and 665 nm. Dashed lines indicate fitting curve traces.**

**
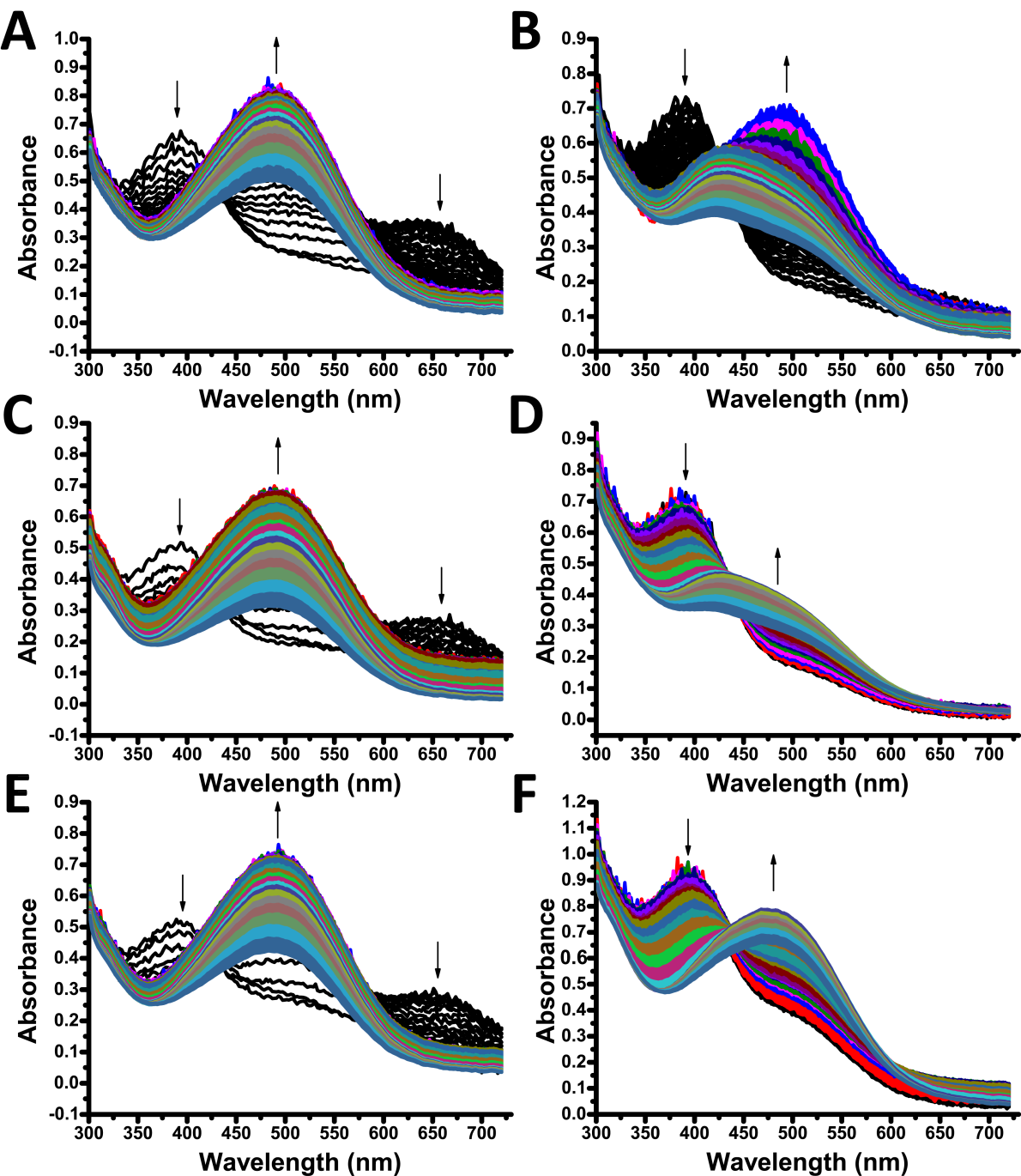
**

**Figure S6: Spectral changes during the reaction of the specified enzyme with nitrocefin in a 1:1 ratio at 5°C. A. Di-Zn(II) VIM-2, pH 5.5. B. Di-Fe(II) VIM-2, pH 5.5. C. Di-Zn(II) VIM-2, pH 6.5. D. Di-Fe(II) VIM-2, pH 6.5. E. Di-Zn(II) VIM-2, pH 7.5. F. Di-Fe(II) VIM-2, pH 7.5.**

**
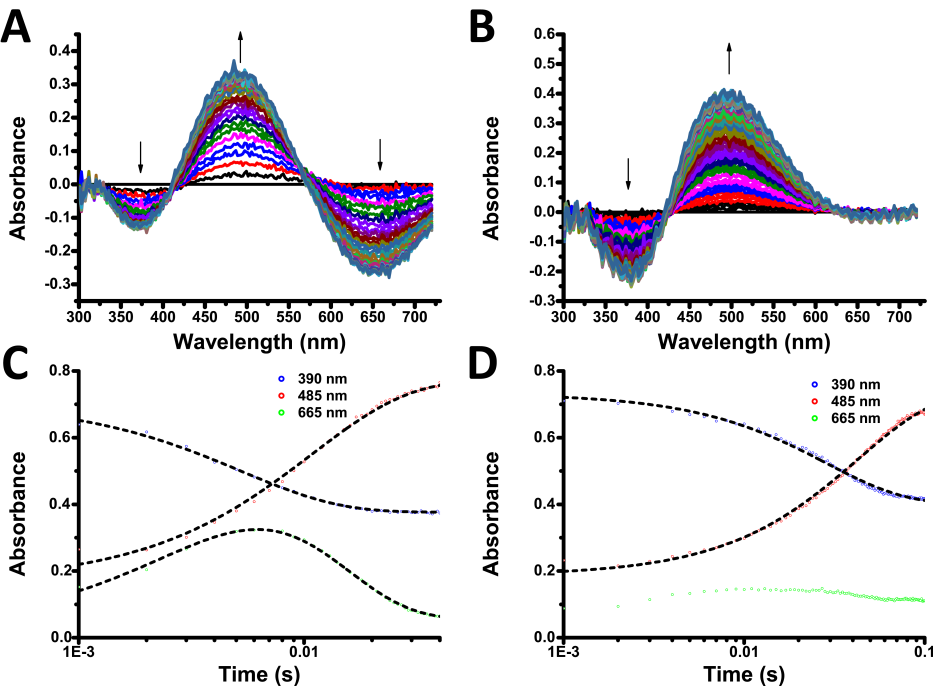
**

**Figure S7: A & B. Spectral changes during the reaction of 50 μM di-Zn(II) VIM-2, or di-Fe(II) VIM-2, respectively, with 50 μM nitrocefin in a 1:1 ratio at pH 5.5 and 5°C. Difference spectra of absorbance across wavelengths 300-750 nm from 0.006–0.04 s using absorbance at 0.006 s as a baseline. Arrows indicate growth or decay of peaks. C & D. Time course of the reaction of 50 μM di-Zn(II) VIM-2, or di-Fe(II) VIM-2, respectively, with 50 μM nitrocefin in a 1:1 ratio of enzyme to substrate at pH 5.5 and 5°C. Absorbance traces at 390, 485 and 665 nm. Dashed lines indicate fitting curve traces.**

**
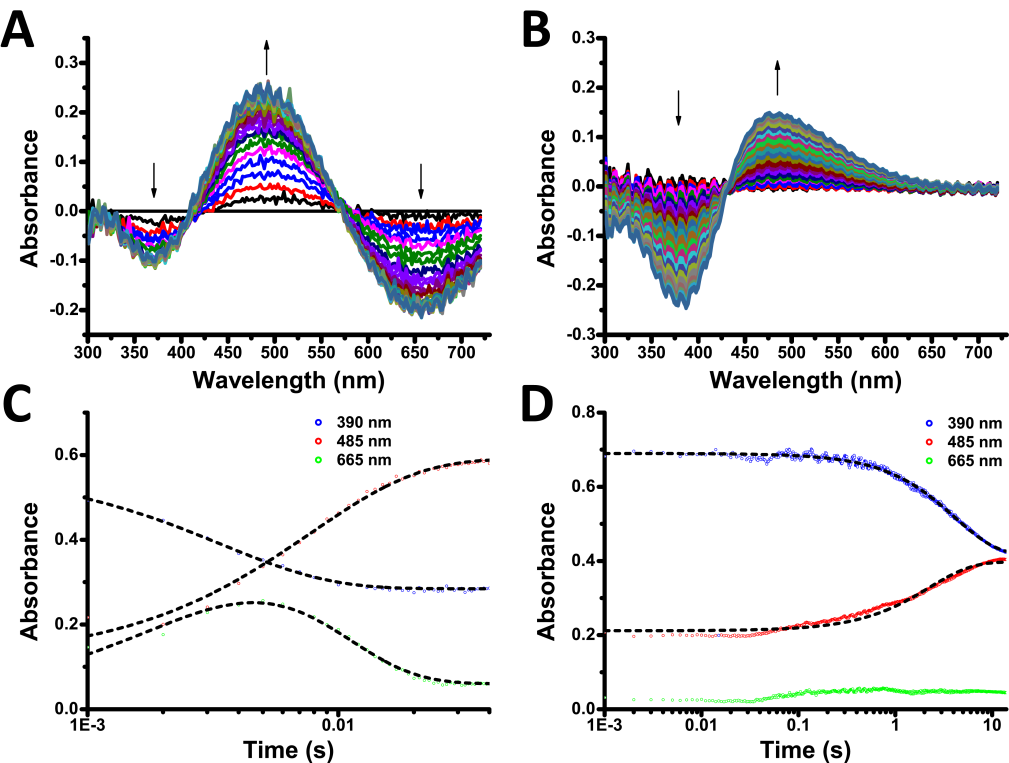
**

**Figure S8: A & B. Spectral changes during the reaction of 50 μM di-Zn(II) VIM-2, or di-Fe(II) VIM-2, respectively, with 50 μM nitrocefin in a 1:1 ratio at pH 6.5 and 5°C. Difference spectra of absorbance across wavelengths 300-750 nm from 0.005–0.04 s using absorbance at 0.005 s as a baseline. Arrows indicate growth or decay of peaks. C & D. Time course of the reaction of 50 μM di-Zn(II) VIM-2, or di-Fe(II) VIM-2, respectively, with 50 μM nitrocefin in a 1:1 ratio of enzyme to substrate at pH 6.5 and 5°C. Absorbance traces at 390, 485 and 665 nm. Dashed lines indicate fitting curve traces.**

**
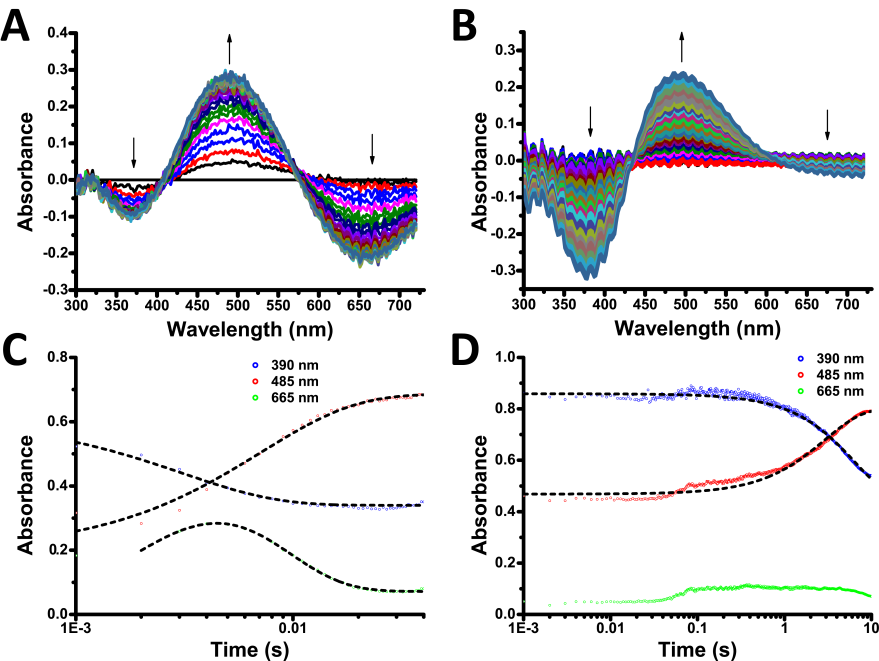
**

**Figure S9: A & B. Spectral changes during the reaction of 50 μM di-Zn(II) VIM-2, or di-Fe(II) VIM-2, respectively, with 50 μM nitrocefin in a 1:1 ratio at pH 7.5 and 5°C. Difference spectra of absorbance across wavelengths 300-750 nm from 0.004–0.04 s using absorbance at 0.004 s as a baseline. Arrows indicate growth or decay of peaks. C & D. Time course of the reaction of 50 μM di-Zn(II) VIM-2, or di-Fe(II) VIM-2, respectively, with 50 μM nitrocefin in a 1:1 ratio of enzyme to substrate at pH 7.5 and 5°C. Absorbance traces at 390, 485 and 665 nm. Dashed lines indicate fitting curve traces.**

**
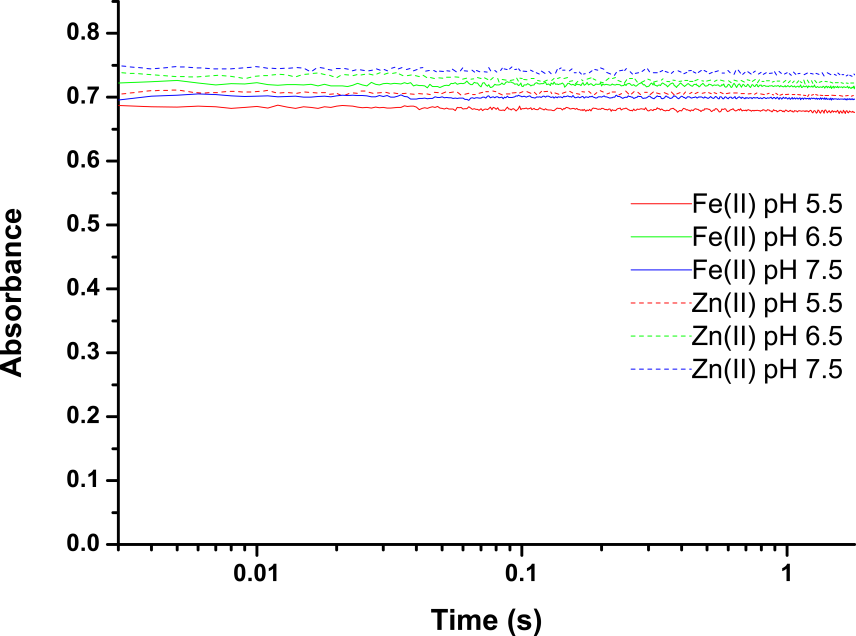
**

**Figure S10: Spectral changes seen at 390 nm when 100 mM nitrocefin is mixed with buffer at pH 5.5, 6.5 or 7.5 supplemented with metal salt. No substrate hydrolysis is seen over the typical time course of an enzyme-catalysed reaction.**

**
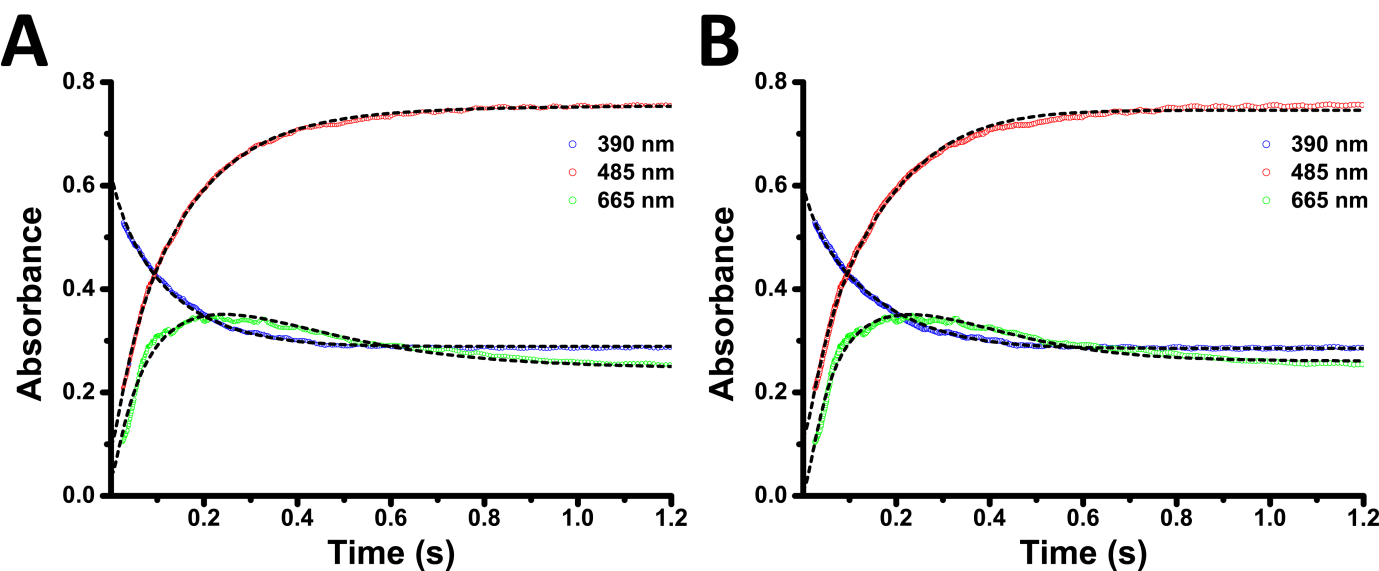
Figure S11: Spectral changes during the reaction of 50 μM di-Fe(II) BcII with 50 μM nitrocefin in a 1:1 ratio at pH 7.5 and 5°C. Dotted lines show the results of data fitting using Kintek Explorer. A. Fitting of data using a linear mechanism. Fitting constants can be found in table S4. B. Fitting of data using a proposed branched mechanism. Fitting constants can be found in table S5 C. Outline of the two mechanisms used to fit the data as well as associated kinetic constants.**

**
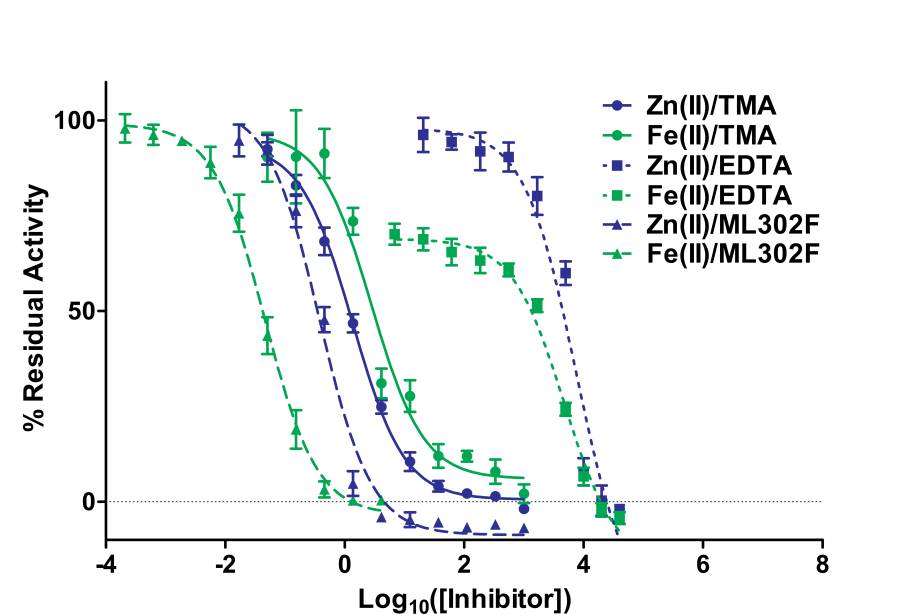
**

**Figure S12: IC_50_ traces for metal-substituted BcII with TMA (circles), EDTA (squares) and ML302F (triangles). Residual hydrolysis of nitrocefin was followed at 485 nm. Blue lines indicate di-Zn(II) BcII and green lines di-Fe(II) BcII.**


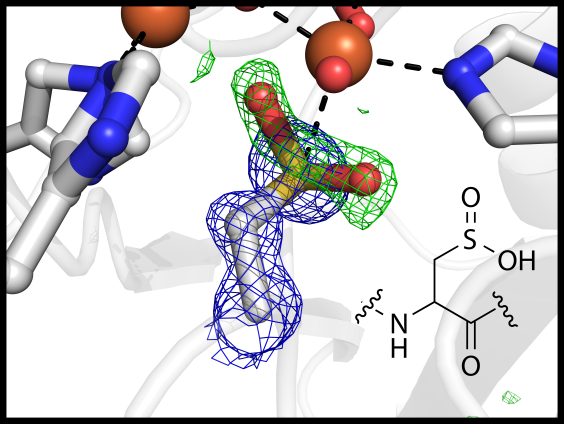


**Figure S13: View of active site residue Cys221 of di-Fe(II)-BcII structure with representative electron density (3.0 σ mFo-DFc OMIT, blue mesh). During refinement additional density seen in the mFo-DFc map (green mesh) was apparent. This was modelled as a doubly-oxidised sulfur (sulfinic acid). Similarly oxidised cysteine residues have been reported with MBLs, e.g. PDB Accession Codes 3I0V and 3I14 [**[**3**](#_ENREF_3)**]. Other minor mFo-DFc differences for this residue observed around Cα were not successfully modelled. Inset the chemical structure of cysteine sulfinic acid as found in the polypeptide chain.**

**References**

1 Mollard, C., Moali, C., Papamicael, C., Damblon, C., Vessilier, S., Amicosante, G., Schofield, C. J., Galleni, M., Frère, J.-M. and Roberts, G. C. K. (2001) Thiomandelic Acid, a Broad Spectrum Inhibitor of Zinc β-Lactamases: KINETIC AND SPECTROSCOPIC STUDIES. J. Biol. Chem. **276**, 45015-45023

2 Poirel, L., Naas, T., Nicolas, D., Collet, L., Bellais, S., Cavallo, J.-D. and Nordmann, P. (2000) Characterization of VIM-2, a Carbapenem-Hydrolyzing Metallo-β-Lactamase and Its Plasmid- and Integron-Borne Gene from a *Pseudomonas aeruginosa* Clinical Isolate in France. Antimicrob. Agents Chemother. **44**, 891-897

3 González, J. M., Buschiazzo, A. and Vila, A. J. (2010) Evidence of Adaptability in Metal Coordination Geometry and Active-Site Loop Conformation Among B1 Metallo-β-Lactamases. Biochemistry. **49**, 7930-7938
